# Rhenium-Sulfido and -Dithiolato Corroles: Reflections on Chalcophilicity

**DOI:** 10.1021/acs.inorgchem.4c04091

**Published:** 2024-12-16

**Authors:** Abraham B. Alemayehu, Nicholas S. Settineri, Arianna E. Lanza, Abhik Ghosh

**Affiliations:** † Department of Chemistry, University of Tromsø, N-9037 Tromsø, Norway; ‡ Advanced Light Source, Lawrence Berkeley National Laboratory, Berkeley, California 94720-8229, United States; § Department of Chemistry, University of Copenhagen, DK-2100 Copenhagen, Denmark

## Abstract

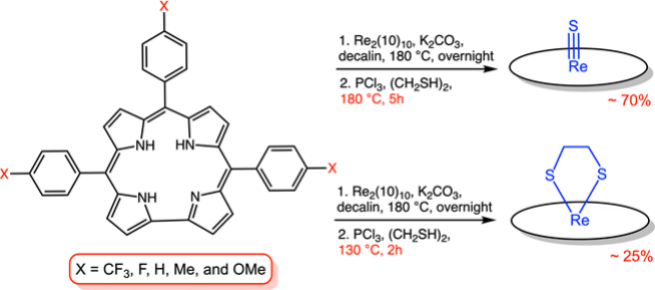

The high-temperature (∼180 °C) reaction between free-base *meso*-triarylcorroles and Re_2_(CO)_10_, followed by exposure to PCl_3_ and thiols (or elemental sulfur), affords rhenium-sulfido (ReS) corroles in 67–76% yields. The use of shorter reaction times, lower temperatures (∼130 °C), and a dithiol (e.g., ethane-1,2-dithiol) also allows the isolation of rhenium-dithiolato corroles, presumptive intermediates on the path to ReS corroles. The ReS corroles exhibit high thermal stability and two reversible oxidations and reductions in their cyclic voltammograms, with redox potentials nearly identical to those observed for analogous ReO corroles. The electrochemical HOMO–LUMO gaps of the complexes, at 2.2 eV, are consistent with ligand-centered oxidation and reduction. The UV–vis spectra of ReS corroles, on the other hand, differ significantly from those of their ReO counterparts. Scalar-relativistic DFT calculations suggest that this difference reflects low-energy LUMO+2 and LUMO+3 levels, consisting of Re–S π-antibonding interactions; the ReO corroles, in contrast, exhibit a larger LUMO+1/LUMO+2 gap, as expected for a relatively classical Gouterman-type metalloporphyrin analogue. The high stability of ReS corroles is consistent with geochemists’ view of rhenium as a moderately chalcophilic element (i.e., one that partitions into sulfide melts) as well as with a recent quantitative analysis of thiophilicity, which indicates that rhenium’s oxophilicity and thiophilicity are essentially evenly balanced.

## Abstract 2

Discovery and synthesis of rhenium-sulfido and rheniumdithiolato corroles, two new structural motifs in the growing family of 5d metallocorroles.

## Introduction^1^

The 5d metallocorroles are fascinating complexes, consisting of a sterically constrained corrole ligand tightly wrapping around a large 5d transition-metal ion.^2,3^ Given the size mismatch between the metal and the macrocyclic ligand, many of the available synthetic routes are capricious and proceed with only low to modest yields.^4−10^ Once synthesized, however, the complexes are typically surprisingly robust. Furthermore, several exhibit long-lived triplet states and efficiently sensitize singlet oxygen formation,^11−13^ promising a variety of applications, perhaps most notably as photosensitizers in photodynamic therapy.^14−17^ Rhenium-oxo^18−23^ and gold^24−31^ corroles are arguably the most promising in this regard, as far as metallocorroles are concerned.

**Chart 1:**
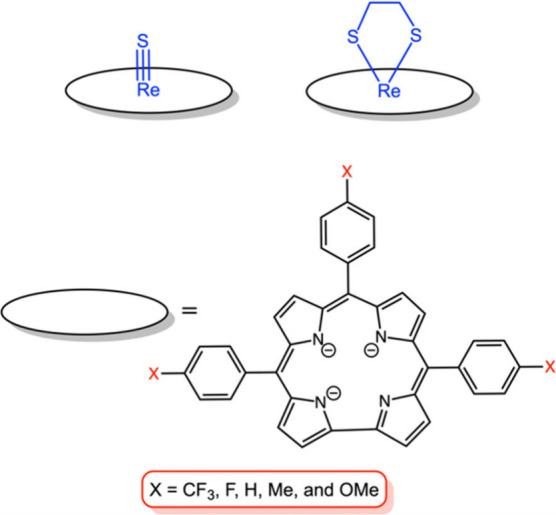
Complexes Synthesized as Part of This Work

Rhenium corroles exhibit a variety of structural motifs. While rhenium-oxo corroles are a key thermodynamic sink, careful manipulation of the synthetic conditions leads variously to rhenium-imido corroles,^32^ metal–metal quadruple-bonded rhenium corrole dimers,^33,34^ and rhenium biscorrole sandwich compounds.^35^ Herein, we report two new structural motifs: rhenium-sulfido corroles and rhenium-dithiolato corroles (Chart 1). The findings add to our appreciation of rhenium as a chalcophilic metal (i.e., a metal that partitions into sulfide melts),^36,37^ which has a strong affinity for not only oxygen but also for sulfur, and potentially also for the heavier chalcogens.

## Results and Discussion

### (a) Discovery and Optimization of Synthetic Methods

Following similar experiments by Bröring and co-workers,^38^ we attempted to activate highly stable ReO *meso*-triarylcorroles with a view to installing axial ligands other than oxide. Accordingly, we exposed an ReO triarylcorroles Re[T*p*XPC](S) [where T*p*XPC refers to the *meso*-tris(*para*-X-phenyl)corrole ligand and X = CH_3_ and OCH_3_] to an excess of PCl_3_, a plausible oxygen atom abstractor, in chlorobenzene at 150 °C under anaerobic conditions, followed by addition of thiophenol. We hoped that an ReCl_2_ corrole intermediate produced in the course of the reaction would ultimately yield Re(SPh)_2_ corrole as the final product. The final product turned out to be different, with a Soret maximum at 391 nm and a higher-energy maximum at 276 nm and a molecular mass corresponding to Re[TpXPC] + 32. Obvious formulations for such a product included Re[T*p*XPC]O_2_, Re[T*p*XPC](PH), and Re[T*p*XPC](S). High-resolution electrospray ionization MS strongly supported the last formulation, i.e., Re[Cor](S), which was ultimately confirmed by 3D electron diffraction^39^ (3D ED) for two complexes, Re[T*p*CH_3_PC](S) and Re[T*p*OCH_3_PC](S).

To develop a general, reproducible, and, ideally, high-yielding synthetic route to ReS corroles, we investigated both elemental sulfur and simple thiols as sources of the sulfido ligand; both proved suitable. Our final, optimized synthesis consists of a one-pot, two-stage protocol. Rhenium insertion was first accomplished via overnight interaction of free-base corrole, H_3_[T*p*XPC], and dirhenium decacarbonyl in the presence of K_2_CO_3_ in decalin at 180 °C. Phosphorus trichloride and ethane-1,2-dithiol were then added, *in that order*, and the reaction was continued for several hours. Upon cooling to room temperature and standard workup and purification, analytically pure products were obtained in yields of 67–76%. The synthesis appears to be rather general, with five different Re[T*p*XPC](S) complexes obtained in comparable yields (X = CF_3_, F, H, CH_3_, and OCH_3_). Importantly, the high-temperature synthesis also underscores the impressive thermal and chemical stability of ReS corroles, which was further confirmed by the stability of the compounds in refluxing decalin over 12 h.

In the course of optimizing the above synthesis, we discovered that shorter reaction times in the second (thiation) stage of the protocol led to a mixture of products, ReO and ReS corroles, indicating incomplete thiation, and rhenium-ethane-1,2-dithiolato corroles, plausible intermediates on the path to ReS corroles. The use of both shorter reaction times (∼2 h) and lower temperatures (∼130 °C) in the thiation step led to the reliable isolation of the rhenium-dithiolato complexes Re[T*p*XPC](C_2_H_4_S_2_) (X = CF_3_, F, H, CH_3_, and OCH_3_), albeit with ReO and ReS corroles as coproducts. The products could be readily separated with column chromatography.

### (b) Proof of Composition and Structure

**Figure 1:**
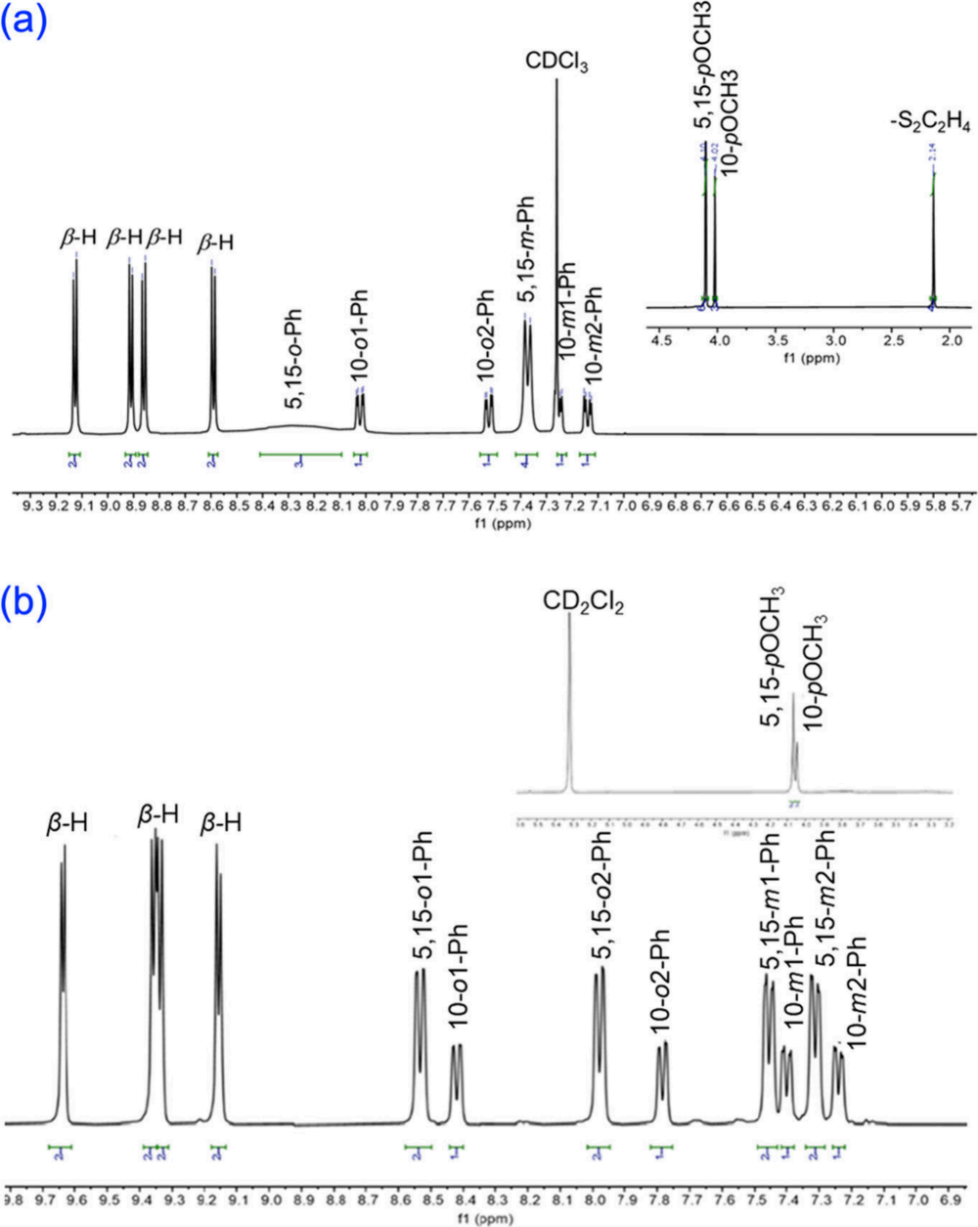
^1^H NMR spectra of (a) Re[T*p*OCH_3_PC](S_2_C_2_H_4_) in CDCl_3_ at 298 K and (b) Re[T*p*OCH_3_PC](S) in CD_2_Cl_2_ at 243 K.

As expected, both the sulfido and dithiolato complexes exhibit diamagnetic ^1^H NMR spectra, similar to those of ReO,^17^ OsN,^5^ and other square-pyramidal metallocorroles.^19−22^ Thus, except for those associated with the 5,15-aryl groups, all peaks proved well-resolved at room temperature. The *ortho* and *meta* protons of the 5,15-aryl groups resolved nicely at 243 K and the full spectra were assigned via 2D NMR analysis. Two exemplary ^1^H NMR spectra are depicted in Figure 1.

**Figure 2:**
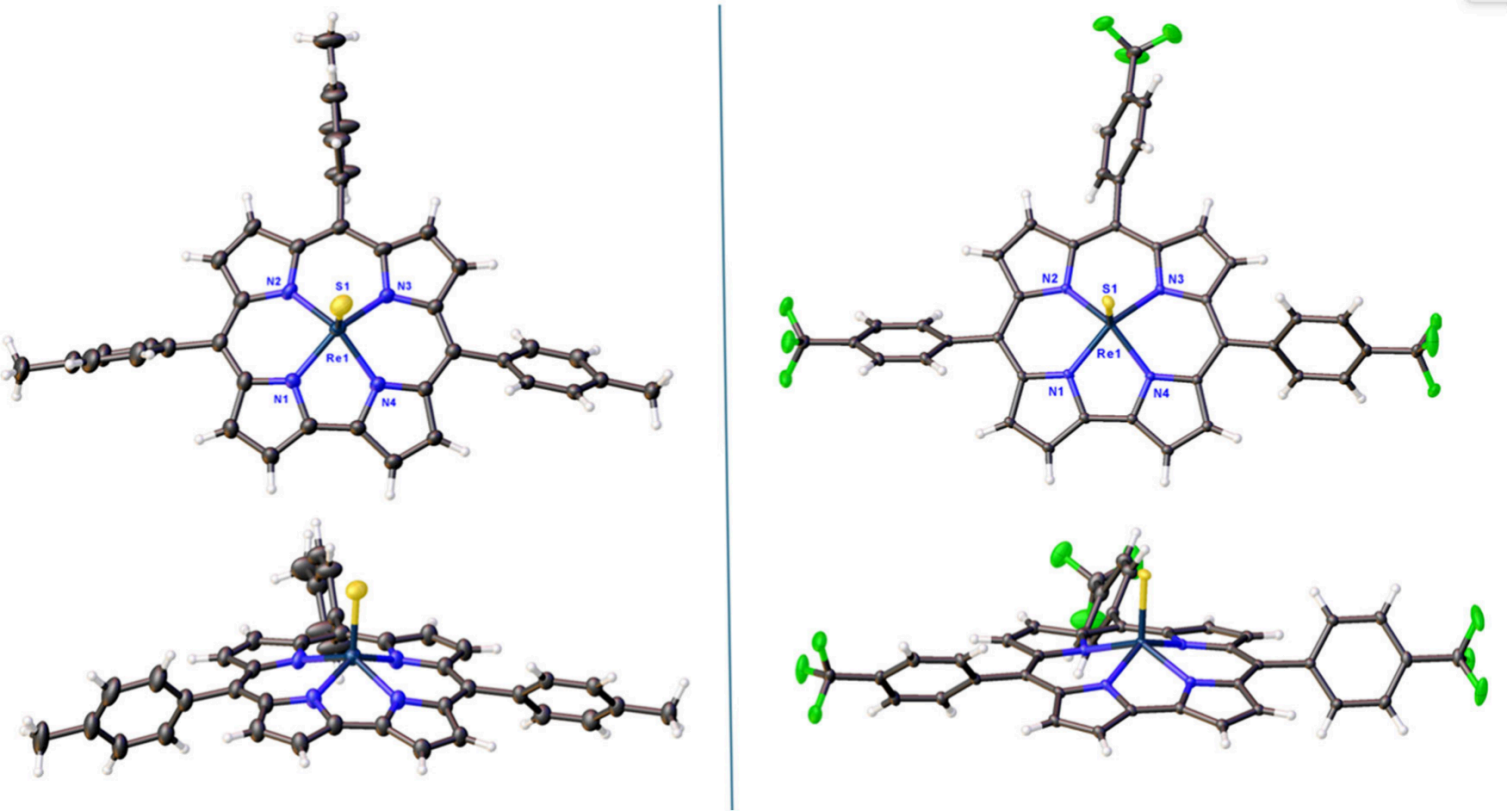
X-ray structures (top and side views) of Re[T*p*CH_3_PC](S) and Re[T*p*CF_3_PC](S). Re[T*p*CH_3_PC](S) (left, Å): Re1–N1 1.988(5), Re1–N2 2.018(4), Re1–N3 1.994(5), Re1–N4 1.987(5), and Re1–S1 2.0752(19). Re[T*p*CF_3_PC](S) (right, Å): Re1–N1 1.986(2), Re1–N2 2.005(2), Re1–N3 2.006(2), Re1–N4 1.979(2), and Re1–S1 2.0766(7).

**Chart 2:**
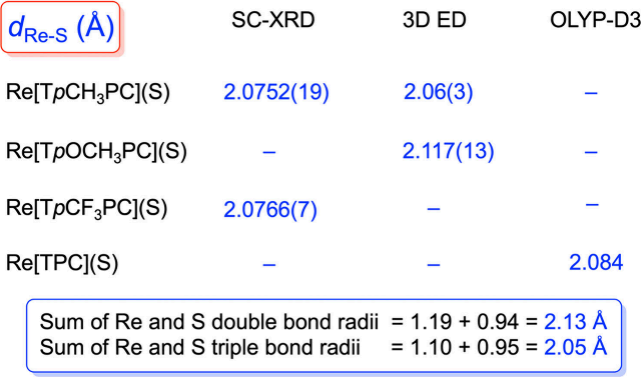
A Comparison of Re-Sulfido Distances (Å) Obtained with Different Methods

**Figure 3:**
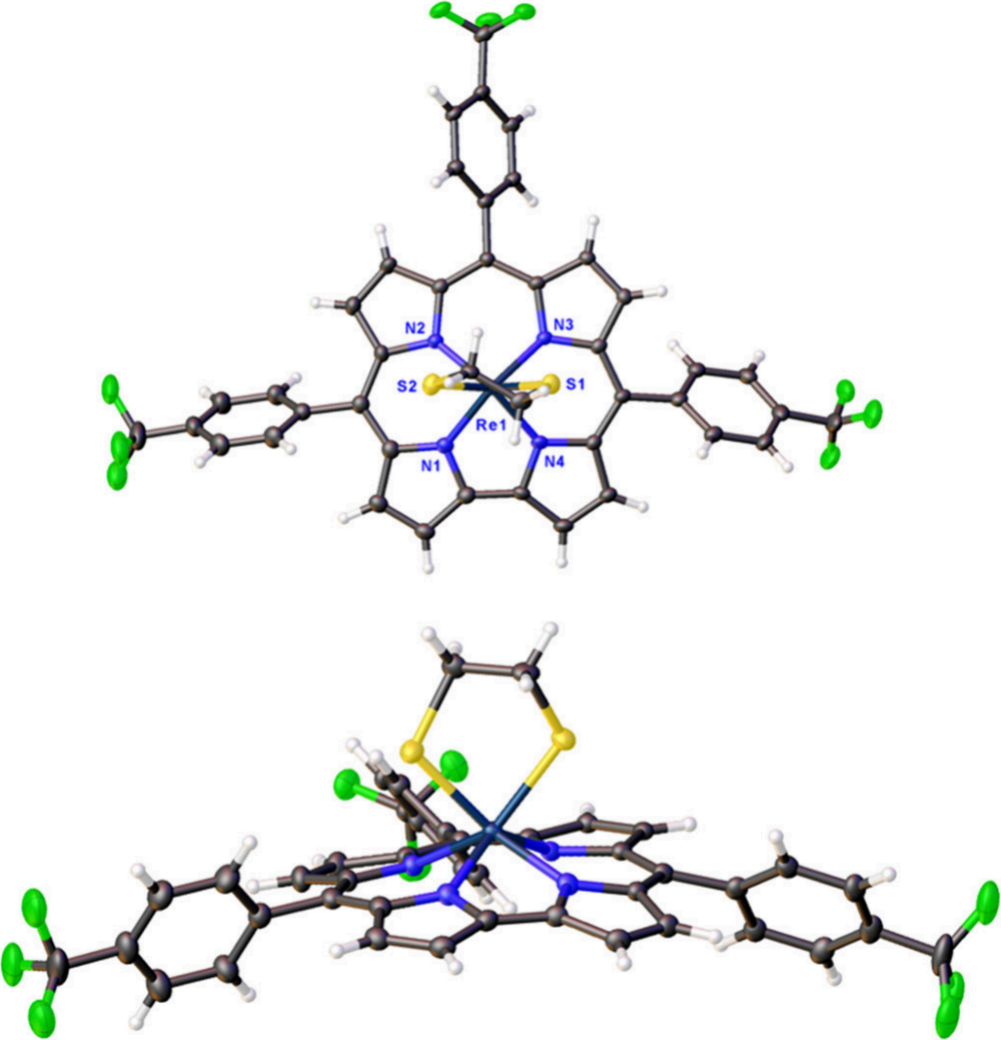
X-ray structure (top and side views) of Re[T*p*CF_3_PC](S_2_C_2_H_4_). Selected bond distances (Å): Re1–N1 2.024(4), Re1–N2 2.047(4), Re1–N3 2.039(4), Re1–N4 2.009(4), Re1–S1 2.2875(14), and Re1–S2 2.3144(15). Thermal ellipsoids are at 30% probability. Positional disorder and solvent molecules have been omitted for clarity.

Crystal structures were obtained for Re[T*p*CH_3_PC](S) and Re[T*p*OCH_3_PC](S) with 3D ED and single-crystal X-ray diffraction (SC-XRD) structures were obtained for Re[T*p*CH_3_PC](S), Re[T*p*CF_3_PC](S), and Re[T*p*CF_3_PC](S_2_C_2_H_4_) (Figures 2 and 3 and Table 1). The 3D ED and XRD structures of Re[T*p*CH_3_PC](S) are in generally excellent agreement, with Re–S distances of 2.06(3) and 2.0752(19) Å, respectively. These values are also consistent with those observed in the SC-XRD structure of Re[T*p*CF_3_PC](S) [2.0766(7)] and the 3D ED structure of Re[T*p*OCH_3_PC](S) [2.117(13)]. For the sulfido complexes, the crystallographic Re–S distances are also in excellent agreement with scalar-relativistic DFT (OLYP^40,41^ -D3^42,43^ /ZORA^44^ -STO-TZ2P as implemented in the ADF program system^45^) optimized geometry of Re[TPC](S) (TPC = *meso*-triphenylcorrole, 2.084 Å), as well as with the sum of Pyykkö’s triple-bond covalent radii for Re and S: 1.10 + 0.95 = 2.05 Å.^46,47^ The sum of the corresponding double bond radii (2.13 Å), on the other hand, appears to be somewhat larger than both the SC-XRD and DFT values, suggesting that the Re–S interactions are best viewed as triple bonds, as indeed expected from elementary ligand field theory considerations. For the reader’s convenience, Chart 2 presents a more visual representation of Re-sulfido distances obtained by different methods. In contrast to the above, the Re–S distances in the dithiolato complex Re[T*p*CF_3_PC](S_2_C_2_H_4_) average around 2.3 Å, the higher value reflecting the lower bond order of the bonds in question. Other structural features of the complexes, such as the average Re–N distances and Re–N_4_ displacements, are unremarkable and very similar to those observed for ReO corroles.^17^

**Table 1:** Crystal Data and Structure Refinement Parameters

Compound	Re[T*p*OCH_3_PC](S)	Re[T*p*CF_3_PC] (S_2_C_2_H_4_)	Re[T*p*CF_3_PC](S)	Re[T*p*CH_3_PC](S)	
CCDC deposition number	2344705	2385196	2385198	2385197	2344704
Method	3D ED	SC-XRD	SC-XRD	3D ED	SC-XRD
Chemical formula	C_40_H_29_N_4_O_3_SRe	C_42_H_24_F_9_N_4_S_2_Re	C_40_H_20_F_9_N_4_SRe	C_40_H_29_N_4_SRe	
Formula weight	831.96	1005.97	988.32	783.93	
Crystal system	Triclinic	Monoclinic	Monoclinic	Monoclinic	
Crystal dimensions	(a)	0.130 × 0.020 × 0.010	0.120 × 0.060 × 0.040	0.0002 × 0.004 × 0.011	0.080 × 0.030 × 0.030
Space group	*P*1̅	*P*2_1_/*n*	*P*2_1_/*c*	*C*2*/c*	
λ (Å)	0.02510	0.7288	0.7288	0.02510	0.7288
*a* (Å)	10.352(6)	15.2995(13)	16.2208(14)	29.905(8)	29.171(2)
*b* (Å)	11.791(7)	8.4238(7)	15.2544(13)	21.407(3)	21.2203(14)
*c* (Å)	16.157(9)	30.564(3)	13.9752(12)	11.191(2)	11.0821(8)
α (deg)	103.48(5)	90	90	90	90
β (deg)	96.62(5)	102.078(4)	94.256(3)	102.489(19)	101.358(3)
γ (deg)	90.16(5)	90	90	90	90
*V* (Å^3^)	1904(2)	3851.9(6)	3448.5(5)	6995(3)	6725.8(8)
*Z*	2	4	4	8	
Temperature (K)	298	100(2)	100(2)	298	100(2)
Density (g/cm^3^)	1.451	1.735	1.904	1.489	1.548
Measured reflections	2862	44835	128751	9232	95091
Unique reflections	1170	7956	13208	3123	8512
Parameters	407	586	514	453	418
Restraints	495	96	18	380	45
*R*_int_	0.3563^a^	0.0785	0.0536	0.1920	0.0598
θ range (deg)	0.046–0.653	1.424–27.212	1.882–34.181	0.042–0.719	1.460–29.408
*R*_1_ [*I* ≥ 2σ(*I*)]	0.1961	0.0536	0.0475	0.1599	0.0630
*wR*_2_ [all data]	0.5161	0.1052	0.0891	0.4456	0.1226
*S* (GooF) all data	1.062	1.029	1.082	1.057	1.024
Max/min residue	0.22/–0.18 (1/Å^2^)	1.33/–1.33 (e/Å^3^)	1.64/-2.80 (e/Å^3^)	0.16/–0.12 (1/Å^2^)	2.75/–3.32 (e/Å^3^)

a Two data sets from two different nanocrystals were merged to increase data completeness.

### (c) UV–Vis and Electrochemical Studies

**Figure 4:**
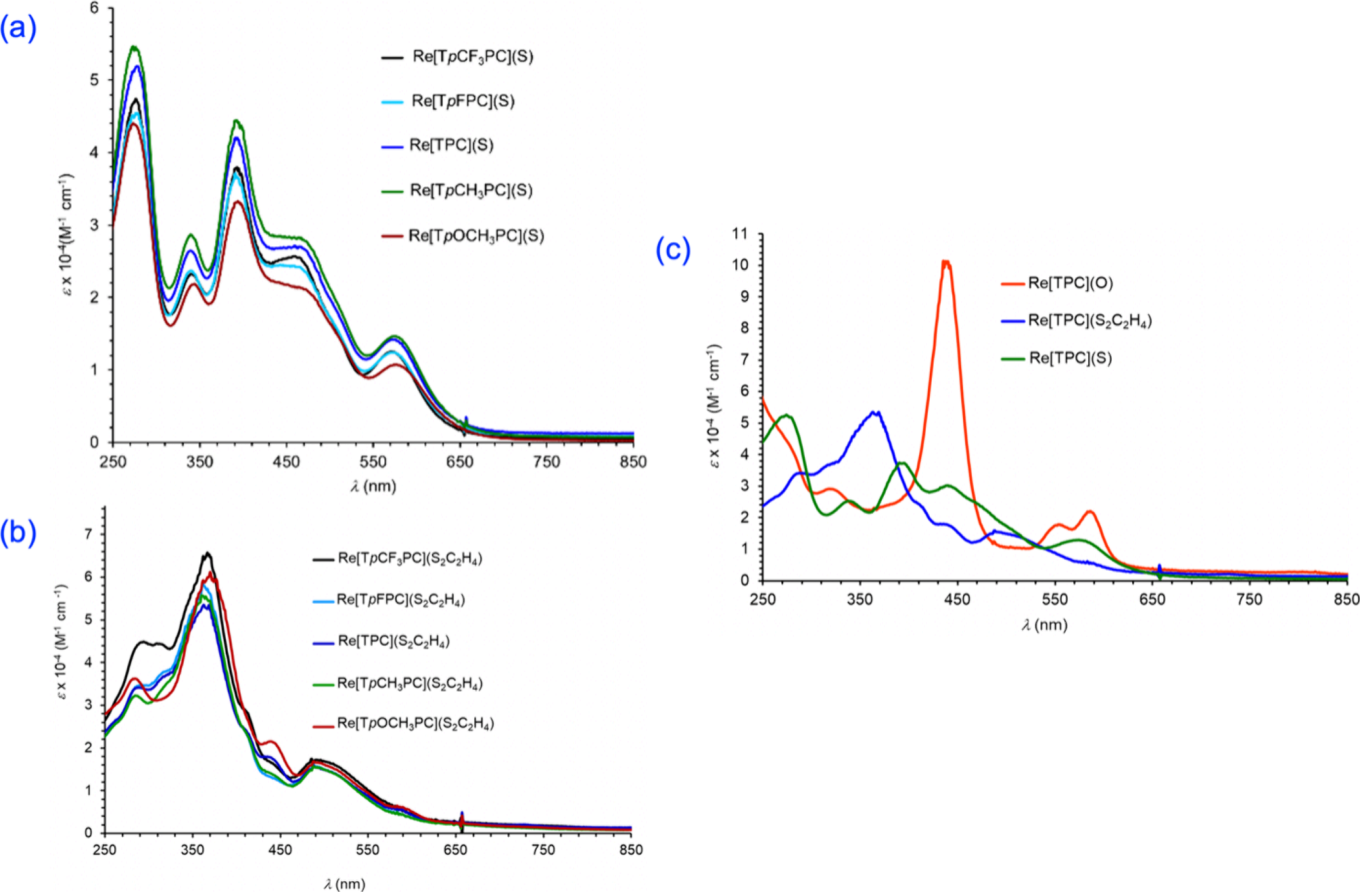
UV–vis spectra of (a) Re[T*p*XPC](S) and (b) Re[T*p*XPC](S_2_C_2_H_4_) in dichloromethane. (c) Comparative UV–vis spectra of Re[TPC](Y) (Y = O, S, S_2_C_2_H_4_). Sample concentrations are in the range 4.0 ± 0.5 mM.

**Figure 5:**
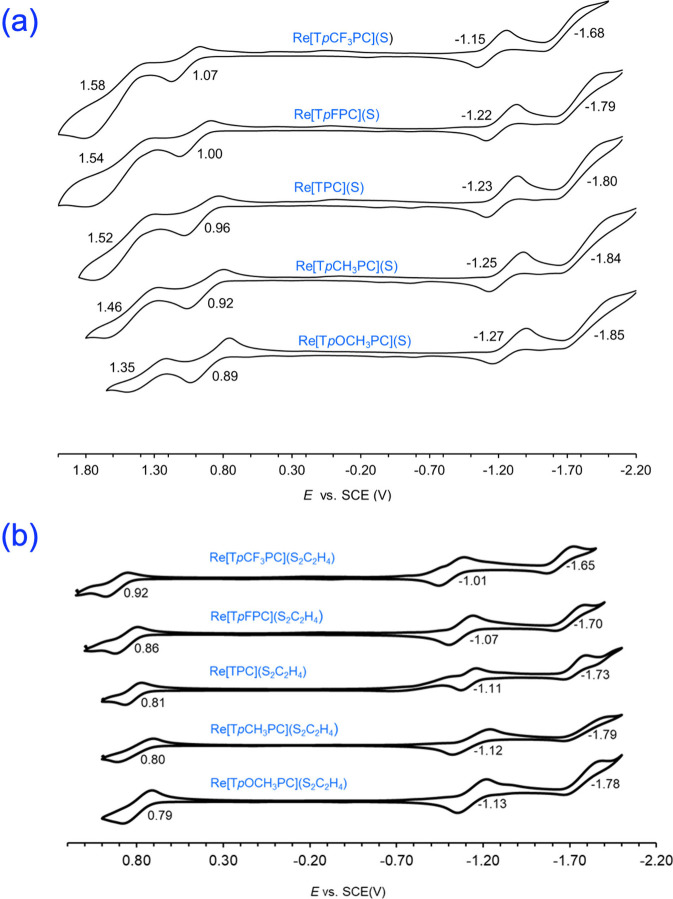
Cyclic voltammograms of (a) Re[T*p*XPC](S) and (b) Re[T*p*XPC](S_2_C_2_H_4_) in 0.1 M solutions of tetrabutylammonium perchlorate in anhydrous dichloromethane; scan rate = 100 mV/s.

Insight into the electronic structure of the new complexes was gathered from UV–vis absorption spectroscopy (Figure 4 and Table 2), cyclic voltammetry (Figure 5 and Table 2), and the aforementioned DFT calculations. Although the new ReS corroles exhibit essentially the same Q-band maxima (573 ± 2 nm) as analogous ReO corroles, indicating near-identical HOMO–LUMO gaps, the overall spectral profiles are quite different, which points to substantial differences in the valence MO architecture. Compared with ReO corroles, the ReS corroles exhibit several broad absorptions in the 250–620 nm range. Thus, significant shoulders are observed for the main Soret feature (392 ± 2 nm) at both lower (275 ± 2 nm) and higher (459 nm) wavelengths. The Re-dithiolato corroles also exhibit numerous broad absorptions in the 250–620 nm region. Interestingly, the lowest-energy maxima of the dithiolato complexes (∼590 nm) are somewhat red-shifted relative to their ReS counterparts (573 ± 2 nm). A wider spread of the absorption features may explain why the main features of the ReS and Re-dithiolato corroles seem weaker than those of their ReO congeners (which presumably exhibit multiple near-coincident absorptions).

**Table 2:** Optical and Electrochemical Properties of Re[T*p*XPC](S), Re[T*p*XPC](O), and Re[T*p*XPC](NPh): λ_max_ (nm) for Soret and Q Bands and *E*_1/2_ Values (V vs. SCE)

Compound	Soret λ_max_	Q λ_max_	*E*1/2(Ox2)	*E*1/2(Ox1)	*E*1/2(red1)	*E*1/2(red2)	Δ*E*	ref
Re[T*p*CF_3_PC](S_2_C_2_H_4_)	294, 309, 367, 410, 441	486, 591	-	0.92	–1.01	–1.65	1.93	This work
Re[T*p*FPC](S_2_C_2_H_4_)	293, 322, 362, 408, 441	487, 589	-	0.86	–1.07	–1.70	1.93	
Re[TPC](S_2_C_2_H_4_)	290, 316, 363, 408, 441	488, 591	-	0.81	–1.11	–1.73	1.92	
Re[T*p*CH_3_PC](S_2_C_2_H_4_)	285, 308, 360, 408, 441	488, 591	-	0.80	–1.12	–1.79	1.92	
Re[T*p*OCH_3_PC](S_2_C_2_H_4_)	284, 307, 370, 408, 441	488, 591	-	0.79	–1.13	–1.78	1.92	
Re[T*p*CF_3_PC](S)	276, 340, 393, 459	571	1.59	1.07	–1.15	–1.68	2.22	
Re[T*p*FPC](S)	276, 341, 391, 459	571	1.54	1.00	–1.22	–1.79	2.22	
Re[TPC](S)	277, 339, 391, 459	572	1.52	0.96	–1.23	–1.80	2.19	
Re[T*p*CH_3_PC](S)	276, 339, 392, 459	575	1.46	0.92	–1.25	–1.84	2.17	
Re[T*p*OCH_3_PC](S)	273, 344, 394, 459	574	1.35	0.89	–1.27	–1.85	2.16	
Re[T*p*CF_3_PC](O)	438	585	-	1.10	–1.16	-	2.26	(17)
Re[T*p*FPC](O)	438	585	-	1.01	–1.23	-	2.24	
Re[TPC](O)	439	585	-	0.98	–1.26	-	2.24	
Re[T*p*CH_3_PC](O)	440	587	-	0.94	–1.29	-	2.23	
Re[T*p*OCH_3_PC](O)	441	592	-	0.93	–1.29	-	2.22	
Re[T*p*CF_3_PC](NPh)	434	577	1.24	0.97	–1.29	-	2.26	(31)
Re[T*p*FPC](NPh)	434	575	1.15	0.88	–1.36	-	2.24	
Re[TPC](NPh)	434	576	1.18	0.86	–1.38	-	2.24	
Re[T*p*CH_3_PC](NPh)	434	578	1.12	0.82	–1.40	-	2.22	
Re[T*p*OCH_3_PC](NPh)	435	578	1.03	0.78	–1.41	-	2.19	

Cyclic voltammetry revealed two reversible oxidations and two reversible reductions for each ReS corrole. The complexes were found to exhibit relatively high first oxidation potentials, 0.89 to 1.07 V, and relatively low first reduction potentials, −1.27 to −1.15 V, vs the SCE, as expected for an electronically innocent corrole macrocycle with a high-valent central metal ion.^48−50^ The electrochemical HOMO–LUMO gap (i.e., the algebraic difference between the first oxidation and reduction potentials) of 2.2 V is essentially the same as that observed for ReO corroles, consistent with the Q bands maxima for ReO and ReS corroles. In contrast, the Re-dithiolato corroles are both ∼100 mV easier to oxidize and ∼100 mV easier to reduce than their ReS counterparts, which translates to a smaller electrochemical HOMO–LUMO gap of ∼2.0 V, suggesting a certain role for Re-dithiolato-based orbitals in the redox processes.

Scalar-relativistic OLYP-D3/ZORA-STO-TZ2P DFT calculations provided a neat explanation for the conundrum that analogous ReO and ReS triarylcorroles exhibit near-identical cyclic voltammograms but substantially different optical spectra. An examination of the Kohn–Sham molecular orbitals indicated that while the four Gouterman-type^51−54^ frontier orbitals are very similar in shape (which are well-established from earlier work^47,55^ and accordingly are not depicted here) and orbital energy for the two classes of complexes, the LUMO+2 and LUMO+3 in the ReS complexes are much lower in energy and closer to the frontier region. These correspond to the two Re–S π* MOs and their low orbital energies account for the relatively complex absorption profile of ReS corroles relative to their relative ReO counterparts. Full photophysical and quantum chemical analyses of ReS corroles will be presented in a follow-up paper on the subject.

### (d) Reflections on Chalcophilicity

Given the wide prevalence of the Re^V^O (rhenyl) and Re^VII^O_3_ (perrhenyl) groups and of the perrhenate anion (ReO_4_^–^), rhenium is often viewed as an oxophilic element by coordination chemists.^56−58^ Geochemists, however, have a somewhat different perspective and regard rhenium as a moderately chalcophilic element (i.e., one that partitions itself into sulfide melts). Thus, in nature, rhenium occurs almost exclusively as part of sulfide minerals such as molybdenite (MoS_2_). A recently developed, quantitative scale of thiophilicity (based on element-chalcogen bond energies) supports the latter view, i.e., rhenium is equally oxophilic and thiophilic. Such a view qualitatively explains the high thermal stability of the ReS corroles reported herein. Scalar-relativistic OLYP-D3/ZORA-STO-TZ2P calculations also support this view. Thus, very similar electronic reaction energies are calculated for the following three reactions (in which Cor denotes unsubstituted corrole):
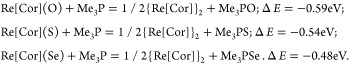


The results indicate that *differences* in rhenium-chalcogen bond energies as a function of the chalcogen are roughly similar to *differences* in phosphine-chalcogen bond energies. Since both ReO corroles^17^ and organophosphine chalcogenides^59,60^ (limiting ourselves to oxygen, sulfur, and selenium among chalcogens) are well-known as stable substances, it makes sense that ReS corroles and as-yet-unknown ReSe corroles should also be so.

## Concluding Remarks

In summary, we have described the discovery and synthesis of rhenium-sulfido and rhenium-dithiolato corroles, two new structural motifs in the growing family of 5d metallocorroles. The ReS corroles exhibit high thermal stability, which may lend itself to applications in photomedicine, especially in photodynamic and photothermal therapies. The findings add to our appreciation of rhenium as a moderately chalcophilic metal, with comparable oxophilic and thiophilic character. We may even envision a new chapter in the coordination chemistry of corroles, in which corroles serve as a privileged platform for stabilizing new 5d-np (*n* > 2) multiple bonds.^61^

## Experimental Section

### Materials and Methods

All chemicals were purchased from Merck, with the notable exception of free-base triarylcorroles, which were prepared according to literature procedures.^62,63^

UV–visible-NIR spectra were recorded on an HP 8453 spectrophotometer. ^1^H NMR spectra were recorded on 400 MHz Bruker Avance III HD spectrometer equipped with a 5 mm BB/1H SmartProbe at 298 K in CDCl_3_ and 243 K in CD_2_Cl_2_ and referenced to residual CHCl_3_ at 7.26 ppm and CH_2_Cl_2_ at 5.31 ppm. High-resolution electrospray ionization mass spectra (HR-ESI-MS) were recorded on an LTQ Orbitrap XL spectrometer in positive mode.

Cyclic voltammetry was carried out at ambient temperature with a Gamry Reference 620 potentiostat equipped with a three-electrode system: a 3 mm disk glassy carbon working electrode, a platinum wire counter electrode, and a saturated calomel reference electrode (SCE). Tetra(*n*-butyl)ammonium hexafluorophosphate was used as the supporting electrolyte. Anhydrous CH_2_Cl _2_ (Aldrich) was used as the solvent. The electrolyte solution was purged with argon for at least 2 min prior to all measurements, which were carried out under an argon blanket. The glassy carbon working electrode was polished using a polishing pad and 0.05 μm polishing alumina from ALS, Japan. All potentials were referenced to the SCE.

### Re[T*p*XPC](S)

To a 50 mL two-necked round-bottom flask fitted with a reflux condenser and containing decalin (15 mL) and a magnetic stirring bar were added a free-base corrole, H_3_[T*p*XPC] (0.19 mmol), Re_2_(CO)_10_ (248.1 mg, 0.38 mmol), and potassium carbonate (150 mg). The contents were deoxygenated with a flow of argon and then refluxed overnight with constant stirring under argon. Phosphorus trichloride (165 μL, 10 equiv) and ethane-1,2-dithiol (160 μL, 10 equiv) were then added and the reaction was continued under reflux (i.e., at ∼180 °C) for ∼5 h. The color of the reaction slowly turned to brown and completion of the reaction was monitored by UV–vis spectroscopy and mass spectrometry. Upon cooling, the reaction mixture was loaded directly on to silica gel column with *n*-heptane as the mobile phase. The decalin was first removed by eluting with pure *n*-heptane. Different solvent mixtures were then used to elute the various ReS corroles: 2:1 *n*-heptane/dichloromethane for X = CF_3_, H, CH_3_ and F; 1:2 *n*-heptane/dichloromethane for X = OCH_3_. All fractions with λ_max_ ∼ 391 nm were collected and evaporated to dryness. The products were further purified with a second round of column chromatography and finally with preparative thin-layer chromatography, all with the same solvent system as in the first round. Yields and analytical details for the different complexes are given below.

### Re[T*p*CF_3_PC](S)

Yield 121.0 mg (0.128 mmol, 67.3%). UV–vis (CH_2_Cl_2_) λ_max_ [nm] (ε x 10^–4^ [M^–1^cm^–1^]): 276 (4.74), 340 (2.32), 393 (3.79), 459 (2.57), 571 (1.25). ^1^H NMR (400 MHz, –30 °C): δ 9.72 (d, 2H, ^3^*J*_HH_ = 4.52 Hz, β-H); 9.36 (d, 2H, ^3^*J*_HH_ = 4.52 Hz, β-H); 9.30 (d, 2H, ^3^*J*_HH_ = 5.0 Hz, β-H); 9.13 (d, 2H, ^3^*J*_HH_ = 5.04 Hz, *β-H*); 8.77 (d, 2H, ^3^*J*_HH_ = 8.56 Hz, 5,15-*o*1-Ph); 8.68 (d, 1H, ^3^*J*_HH_ = 8.44 Hz, 10-*o*1-Ph); 8.24—8.15 (overlapping doublets, 5H, 5,10,15-*m*1-Ph and 5,15-o2-Ph); 8.08 (d, 2H, ^3^*J*_HH_ = 8.20 Hz, 5,15*-m*2-Ph); 8.02 (s, 2H, 10*-o*2 and 10-*m*2-Ph). MS (ESI): M^+^ = 946.0819 (expt), 946.0817 (calcd for C_40_H_20_N_4_F_9_SRe).

### Re[T*p*FPC](S)

Yield 115.1 mg (0.145 mmol, 76.1%). UV–vis (CH_2_Cl_2_): λ_max_ [nm] (ε x 10^–4^ [M^–1^cm^–1^]): 276 (4.54), 341 (2.36), 391 (3.71), 459 (2.43), 571 (1.24). ^1^H NMR (400 MHz, –30 °C): δ 9.68 (d, 2H, ^3^*J*_HH_ = 4.52 Hz, β-H); 9.36 (d, 2H, ^3^*J*_HH_ = 4.52 Hz, β-H); 9.31 (d, 2H, ^3^*J*_HH_ = 4.88 Hz, β-H); 9.13 (d, 2H, ^3^*J*_HH_ = 4.88 Hz, β-H); 8.60 (m, 2H, 5,15-*o*1-Ph); 8.50 (m, 1H, 10-*o*1-Ph); 8.01 (m, 2H, 5,15-*o*2-Ph); 7.83 (m, 1H, 10-*o*2-Ph); 7.68—7.56 (m, 3H, 5,10,15-*m*1-Ph); 7.51 (m, 2H, 5,15-*m*2-Ph); 7.44 (m, 1H, 10-*m*2-Ph). MS (ESI): M^+^ = 796.0921 (expt), 796.0913 (calcd for C_37_H_20_F_3_N_4_SRe).

### Re[TPC](S)

Yield 104.0 mg (0.140 mmol, 73.8%). UV–vis (CH_2_Cl_2_): λ_max_ [nm] (ε x 10^–4^ [M^–1^cm^–1^]): 277 (5.19), 339 (2.64), 391 (4.21), 459 (2.71), 572 (1.42). ^1^H NMR (400 MHz, –30 °C): δ 9.71 (d, 2H, ^3^*J*_HH_ = 4.52 Hz, β-H); 9.41 (d, 2H, ^3^*J*_HH_ = 4.52 Hz, β-H); 9.37 (d, 2H, ^3^*J*_HH_ = 4.80 Hz, β-H); 9.18 (d, 2H, ^3^*J*_HH_ = 4.88 Hz, β-H); 8.66 (d, 2H, ^3^*J*_HH_ = 7.32 Hz, 5,15-*o*1-Ph); 8.56 (d, 1H, ^3^*J*_HH_ = 7.48 Hz, 10-*o*1-Ph); 8.10 (d, 2H, ^3^*J*_HH_ = 6.96 Hz, 5,15-*o*2-Ph); 7.98 (t, 2H, ^3^*J*_HH_ = 7.48 Hz, 5,15-*m*1-Ph); 7.91 (m, 2H, 10-*o*2-Ph and 10-*m*1-Ph); 7.84 (m, 5H, 5,15-*m*2-Ph and 5,10,15-*p*Ph); 7.77 (t, 1H, ^3^*J*_HH_ = 7.44 Hz, 10-*m*2-Ph). MS (ESI): M^+^ = 742.1197 (expt), 742.1195 (calcd for C_37_H_23_N_4_SRe).

### Re[T*p*CH_3_PC](S)

Yield 108.0 mg (0.138 mmol, 72.5%). UV–vis (CH_2_Cl_2_): λ_max_ [nm] (ε x 10^–4^ [M^–1^cm^–1^]): 276 (5.46), 308 (3.16), 339 (2.86), 392 (4.44), 459 (2.83); 575 (1.46). ^1^H NMR (400 MHz, –30 °C): δ 9.63 (d, 2H, ^3^*J*_HH_ = 4.4 Hz, β-H); 9.35 (d, 2H, ^3^*J*_HH_ = 4.4 Hz, β-H); 9.32 (d, 2H, ^3^*J*_HH_ = 4.8 Hz, β-H); 9.13 (d, 2H, ^3^*J*_HH_ = 5.0 Hz, β-H); 8.49 (d, 2H, ^3^*J*_HH_ = 7.72 Hz, 5,15-*o*1-Ph); 8.38 (d, 1H, ^3^*J*_HH_ = 7.56 Hz, 10-*o*1-Ph); 7.92 (d, 2H, ^3^*J*_HH_ = 7.60 Hz, 5,15-*o*2-Ph); 7.73 (d, 3H, ^3^*J*_HH_ = 7.68 Hz, 5,15-m1-Ph and 10-*o*2-Ph); 7.68 (d, 1H, ^3^*J*_HH_ = 6.36 Hz, 10-*m*1-Ph); 7.58 (d, 2H, ^3^*J*_HH_ = 7.84 Hz, 5,15-*m*2-Ph); 7.51 (d, 1H, ^3^*J*_HH_ = 7.68 Hz, 10-*m*2-Ph); 2.68 (s, 6H, 5,15-*p*-CH_3_); 2.66 (s, 3H, 10-*p*-CH_3_). MS (ESI): M^+^ = 784.1665 (expt), 784.1665 (calcd for C_40_H_29_N_4_SRe).

### Re[T*p*OCH_3_PC](S)

Yield 112.0 mg (0.135 mmol, 70.8%). UV–vis (CH_2_Cl_2_): λ_max_ [nm] (ε x 10^–4^ [M^–1^cm^–1^]): 273 (4.40), 307 (3.11), 344 (2.18), 394 (3.32), 459 (2.15); 574 (1.07). ^1^H NMR (400 MHz, –30 °C): δ 9.64 (d, 2H, ^3^*J*_HH_ = 4.4 Hz, β-H); 9.36 (d, 2H, ^3^*J*_HH_ = 4.52 Hz, β-H); 9.34 (d, 2H, ^3^*J*_HH_ = 4.96 Hz, β-H); 9.15 (d, 2H, ^3^*J*_HH_ = 5.0 Hz, β-H); 8.53 (dd, 2H, ^3^*J*_HH_ = 8.32, 2.32 Hz, 5,15-*o*1-Ph); 8.42 (dd, 1H, ^3^*J*_HH_ = 8.32, 2.32 Hz, 10-*o*1-Ph); 7.98 (dd, 2H, ^3^*J*_HH_ = 8.32, 2.20 Hz, 5,15-*o*2-Ph); 7.78 (dd, 1H, ^3^*J*_HH_ = 8.28, 2.36 Hz, 10-*o*2-Ph); 7.45 (dd, 2H, ^3^*J*_HH_ = 8.44, 2.80 Hz, 5,15-*m*1-Ph); 7.40 (dd, 1H, ^3^*J*_HH_ = 8.44, 2.80 Hz, 10-*m*1-Ph); 7.31 (dd, 2H, ^3^*J*_HH_ = 8.44, 2.92 Hz, 5,15-*m*2-Ph); 7.24 (dd, 1H, ^3^*J*_HH_ = 8.44, 2.92 Hz, 10-*m*2-Ph); 4.07 (s, 6H, 5,15*-p*-OCH_3_); 4.05 (s, 3H, 10-*p*-OCH_3_). MS (ESI): M^+^ = 832.1518 (expt), 832.1513 (calcd for C_40_H_29_O_3_N_4_SRe).

### General Procedure for the Synthesis of Re[T*p*XPC](S_2_C_2_H_4_)

To a 50 mL two-necked round-bottom flask fitted with a reflux condenser and containing decalin (15 mL) and a magnetic stirring bar were added a free-base corrole, H_3_[T*p*XPC] (0.096 mmol), Re_2_(CO)_10_ (125.3 mg, 0.192 mmol), and potassium carbonate (100 mg). The contents were deoxygenated with a flow of argon and then refluxed overnight with constant stirring under argon. Upon lowering the reaction temperature to ∼130 °C, phosphorus trichloride (165 μL, 10 equiv) and ethane-1,2-dithiol (160 μL, 10 equiv) were added and the reaction continued for an additional 2 h. Mass spectrometric analysis of the reaction mixture indicated the presence of a mixture of products, including ReO, ReS, and Re-thiolato corroles. Heating was stopped at that point so as to intercept the Rethiolato corroles, the presumptive intermediates on the way to ReS corroles. Upon cooling to room temperatue, the reaction mixture was loaded directly on to silica gel column with *n*-heptane as the mobile phase. Decalin was first removed by eluting with pure heptane. Different solvent mixtures were then used to elute the various mixtures. The wine-red ReO corroles eluted first with 3:1 *n*-heptane/dichloromethane, followed by brown ReS corroles, which were eluted with 2:1 *n*-heptane/dichloromethane. Finally, the Re-dithiolato corroles were eluted with pure dichloromethane. The latter were further purified by preparative thin layer using 1:1 *n*-heptane/dichloromethane as the mobile phase. The brown final products (λ_max_ ∼ 363 nm) were collected and fully characterized, as detailed below.

### Re[T*p*CF_3_PC](S_2_C_2_H_4_)

Yield 22.1 mg (0.022 mmol, 22.9%). UV–vis (CH_2_Cl_2_) λ_max_ (nm) (ε x 10^–4^ [M^–1^cm^–1^]): 294 (4.49), 309 (4.44), 367 (6.57), 410 (2.89), 441 (1.65), 486 (1.74). ^1^H NMR (400 MHz, 25 °C): δ 9.21 (d, 2H, ^3^*J*_HH_ = 4.64 Hz, β-H); 8.89 (d, 2H, ^3^*J*_HH_ = 4.64 Hz, β-H); 8.81 (d, 2H, ^3^*J*_HH_ = 4.88 Hz, *β*-H); 8.53 (d, 2H, ^3^*J*_HH_ = 5.04 Hz, *β-H*); 8.26 (d, 1H, ^3^*J*_HH_ = 8.80 Hz, 10-*o*1-Ph); 8.13 (d, 4H, ^3^*J*_HH_ = 8.04 Hz, 5,15-*m-*Ph); 8.02 (d, 1H, ^3^*J*_HH_ = 8.80 Hz 10-*m*1-Ph); 7.90 (d, 1H, ^3^*J*_HH_ = 8.04 Hz, 10*-o*2-Ph); 7.73 (d, 1H, ^3^*J*_HH_ = 7.84 Hz, 10-*m*2-Ph); 2.20 (s, 4H, S_2_C_2_H_4_). MS (ESI): M^+^ = 1006.0851 (expt), 1006.0849 (calcd for C_42_H_24_N_4_F_9_S_2_Re).

### Re[T*p*FPC](S_2_C_2_H_4_)

Yield 20.5 mg (0.024 mmol, 24.9%). UV–vis (CH_2_Cl_2_) λ_max_ (nm) (ε x 10^–4^ [M^–1^cm^–1^]): 293 (3.47), 322 (3.82), 362 (5.83), 408 (2.45), 441 (1.29), 487 (1.60). ^1^H NMR (400 MHz, 25 °C): δ 9.17 (d, 2H, ^3^*J*_HH_ = 4.64 Hz, *β*-H); 8.89 (d, 2H, ^3^*J*_HH_ = 4.64 Hz, *β*-H); 8.83 (d, 2H, ^3^*J*_HH_ = 4.88 Hz, *β*-H); 8.55 (d, 2H, ^3^*J*_HH_ = 5.04 Hz, *β-H*); 8.08 (ddd, 1H, ^3^*J*_HH_ = 7.93, 5.36, 2.32 Hz, 5,15-*o*1-Ph); 7.55 (m, 8H, 5,15-*o*2-Ph, 5,15-*m*Ph, and 10-*o*1-Ph); 7.44 (td, 1H, ^3^*J*_HH_ = 8.56, 2.74 Hz, 10-*m*1-Ph); 7.32 (td, 1H, ^3^*J*_HH_ = 8.50, 2.74 Hz, 10*-m*2-Ph); 2.16 (s, 4H, S_2_C_2_H_4_). MS (ESI): M^+^ = 856.0943 (expt), 856.0945 (calcd for C_39_H_24_N_4_F_3_S_2_Re).

### Re[TPC](S_2_C_2_H_4_)

Yield 19.2 mg (0.024 mmol, 24.9%). UV–vis (CH_2_Cl_2_) λ_max_ (nm) (ε x 10^–4^ [M^–1^cm^–1^]): 290 (3.41), 317 (3.68), 363 (5.35), 408 (2.47), 441 (1.75), 488 (1.59). ^1^H NMR (400 MHz, 25 °C): δ 9.15 (d, 2H, ^3^*J*_HH_ = 4.64 Hz, *β*-H); 8.91 (d, 2H, ^3^*J*_HH_ = 4.52 Hz, β-H); 8.83 (d, 2H, ^3^*J*_HH_ = 4.88 Hz, β-H); 8.56 (d, 2H, ^3^*J*_HH_ = 4.88 Hz, *β-H*); 8.11 (d, 1H, ^3^*J*_HH_ = 8.80 Hz, 10-*o*1-Ph); 7.88—7.64 (m, 9H, Ph); 7.60 (d, 1H, ^3^*J*_HH_ = 5.00 Hz 10-*m*2-Ph); 2.16 (s, 4H, S_2_C_2_H_4_). MS (ESI): M^+^ = 802.1233 (expt), 802.1228 (calcd for C_39_H_27_N_4_S_2_Re).

### Re[T*p*CH_3_PC](S_2_C_2_H_4_)

Yield 21.6 mg (0.026 mmol, 26.7%). UV–vis (CH_2_Cl_2_) λ_max_ (nm) (ε x 10^–4^ [M^–1^cm^–1^]): 285 (3.23), 360 (5.57),408 (2.45), 441 (1.38), 488 (1.57). ^1^H NMR (400 MHz, 25 °C): δ 9.13 (d, 2H, ^3^*J*_HH_ = 4.64 Hz, *β*-H); 8.91 (d, 2H, ^3^*J*_HH_ = 4.52 Hz, *β*-H); 8.84 (d, 2H, ^3^*J*_HH_ = 4.88 Hz, *β*-H); 8.58 (d, 2H, ^3^*J*_HH_ = 4.88 Hz, *β-H*); 8.18 (s, 4H, 5,15-*o*-Ph); 7.99 (d, 1H, ^3^*J*_HH_ = 7.80 Hz, 10-*o1-*Ph); 7.65 (d, 4H, ^3^*J*_HH_ = 7.92 Hz 5,15-*m*-Ph); 7.53 (d, 1H, ^3^*J*_HH_ = 7.84 Hz, 10*-m*1-Ph); 7.49 (d, 1H, ^3^*J*_HH_ = 7.72 Hz, 10-*o*2-Ph); 7.41 (d, 1H, ^3^*J*_HH_ = 7.84 Hz, 10-*m*2-Ph); 2.72 (s, 6H, 5,15-*p*CH_3_); 2.62 (s, 3H, 10-*p*CH_3_); 2.15 (s, 4H, S_2_C_2_H_4_). MS (ESI): M^+^ = 844.1697 (expt), 844.1697 (calcd for C_42_H_33_N_4_S_2_Re).

### Re[T*p*OCH_3_PC](S_2_C_2_H_4_)

Yield 20.3 mg (0.023 mmol, 23.7%). UV–vis (CH_2_Cl_2_) λ_max_ (nm) (ε x 10^–4^ [M^–1^cm^–1^]): 284 (3.62), 370 (6.12),408 (2.92), 441 (2.14), 488 (1.68). ^1^H NMR (400 MHz, 25 °C): δ 9.13 (d, 2H, ^3^*J*_HH_ = 4.64 Hz, *β*-H); 8.91 (d, 2H, ^3^*J*_HH_ = 4.52 Hz, *β*-H); 8.86 (d, 2H, ^3^*J*_HH_ = 4.88 Hz, *β*-H); 8.59 (d, 2H, ^3^*J*_HH_ = 4.92 Hz, *β-H*); 8.29 (s, 4H, 5,15-*o*-Ph); 8.02 (dd, 1H, ^3^*J*_HH_ = 8.44, 2.32 Hz, 10-*o1-*Ph); 7.52 (dd, 4H, ^3^*J*_HH_ = 8.44, 2.32 Hz 5,15-*m*-Ph); 7.37 (d, 1H, ^3^*J*_HH_ = 9.12 Hz, 10*-o*2-Ph); 7.24 (dd, 1H, ^3^*J*_HH_ = 8.44, 2.80 Hz, 10-*m*2-Ph); 7.14 (dd, 1H, ^3^*J*_HH_ = 8.44, 2.68 Hz, 10-m2-Ph); 4.10 (s, 6H, 5,15-*p*OCH_3_); 4.02 (s, 3H, 10-*p*OCH_3_); 2.14 (s, 4H, S_2_C_2_H_4_). MS (ESI): M^+^ = 892.1548 (expt), 892.1545 (calcd for C_42_H_33_N_4_O_3_S_2_Re).

### Sample Preparation for Crystallography

Crystals were grown by slow diffusion of methanol into concentrated solutions of the samples in dichloromethane.

### 3D Electron Diffraction (3D ED)

A drop of a suspension containing microcrystals was deposited on a microscopy glass slide and allowed to evaporate in air. Once the mother liquor had dried, polarized light microscopy was used to verify the crystallinity of the solid deposit. The solid was then gently scraped from the glass and deposited onto a 300-mesh copper TEM grid coated with a continuous film of ultrathin amorphous carbon (from Electron Microscopy Sciences). 3D electron diffraction measurements were performed at ambient temperature and high vacuum on a Rigaku XtaLAB Synergy-ED electron diffractometer,^64^ equipped with a lanthanum hexaboride (LaB_6_) electron source operating at 200 kV (λ = 0.0251 Å). Diffraction patterns were collected with a Rigaku HyPix-ED detector with a scan width of 0.5° during continuous rotation of the crystals over ∼120°. Diffraction data were collected on five crystals for each complex using the CrysAlisPro^65^ program. The best diffracting crystals were platelets a few microns wide and less than 0.5 μm thick. Selected area apertures (corresponding to either 2 or 1 μm diameter) were used to collect data from thin regions of the crystals. Data processing was carried out with PETS 2.0^66^ and CrysAlisPro.^64^ For Re[T*p*CH_3_PC](S), the structure was solved from a single data set with 85% completeness. Due to the lower crystallographic symmetry of Re[T*p*OCH_3_PC](S), the integrated intensities of two crystals were merged with CrysAlisPro to achieve a completeness of 95.4%.

The crystal structures were solved ab initio with SHELXT^67^ and OLEX2^68^ and the positions of all non-hydrogen atoms could be determined and refined with anisotropic displacement parameters (ADP). Least-squares refinement in kinematic approximation was performed with the programs SHELXL^69^ and ShelXle^70^ using scattering factors for electrons.^71^ An extinction coefficient was refined to mitigate the effect of multiple diffraction. Because of the limited resolution of the diffraction data, soft restraints were applied to preserve the geometry of the substituents and the consistency of ADPs. Hydrogen atoms were placed in geometrically idealized positions and refined with a riding model. The structures feature large void channels (10–18% of the unit cell volume) but no solvent molecules were located in the voids; they were probably completely removed under the high vacuum conditions in the electron diffractometer.

### Single-Crystal X-ray Diffraction Analyses

X-ray data were collected on beamline 12.2.1 at the Advanced Light Source, Lawrence Berkeley National Laboratory. Samples were mounted on MiTeGen Kapton loops and placed in a 100(2) K nitrogen cold stream provided by an Oxford Cryostream 800 Plus low-temperature apparatus on the goniometer head of a Bruker D8 diffractometer equipped with a PHOTON II CPAD detector operating in shutterless mode. Diffraction data were collected using synchrotron radiation monochromated using silicon(111) to a wavelength of 0.7288(1) Å. An approximate full-sphere of data was collected using a combination of φ and ω scans with scan speeds of one second per degree. The structures were solved by intrinsic phasing (SHELXT^66^) and refined by full-matrix least-squares on F^2^ (SHELXL-2014^68^). All non-hydrogen atoms were refined anisotropically. Hydrogen atoms were geometrically calculated and refined as riding atoms. A solvent mask was applied within OLEX2 to the Re[T*p*CH_3_PC](S) and Re[T*p*CF_3_PC](S_2_C_2_H_4_) structures due to highly disordered or ill-defined solvent molecules in the crystal structures.

### DFT Calculations

Key details of the DFT calculations have already been indicated in the discussion above. In addition, the calculations were carried out with a spin-unrestricted formalism, a scalar-relativistic ZORA (Zeroth Order Regular Approximation to the Dirac equation) Hamiltonian, all-electron ZORA STO-TZ2P basis sets, fine integration grids, and carefully tested, tight criteria for SCF and geometry optimization cycles.

## Data Availability

All data generated or analyzed in this study are included in this published article and its Supporting Information.

## Supporting Information Available

ESI mass spectra, selected photophysical data, and optimized DFT coordinates (PDF)
