# Supplementary material for: Rhenium-Sulfido and -Dithiolato Corroles: Reflections on Chalcophilicity
Source: Inorg Chem. 2024 Dec 16;63(52):24787–96. doi: 10.1021/acs.inorgchem.4c04091 (PMC11688660; doi:10.1021/acs.inorgchem.4c04091)
Supplement: Supplementary file 1 — ic4c04091_si_001.pdf [file ic4c04091_si_001.pdf]

Supporting Information

Rhenium-Sulfido and -Dithiolato Corroles: Reflections on Chalcophilicity

Abraham B. Alemayehu,[a] Nicholas S. Settineri,[b] Arianna E. Lanza,[c] and Abhik Ghosh*,[a]

[a]Department of Chemistry, University of Tromsø, N-9037 Tromsø, Norway

[b]Advanced Light Source, Lawrence Berkeley National Laboratory, Berkeley, CA 94720-8229, United States.

[c]Department of Chemistry, University of Copenhagen, DK-2100 Copenhagen, Denmark

Table of contents

A. ^{1}H NMR spectra S2

B. HR-ESI mass spectra S12

C. OLYP-D3 optimized coordinates S20

A. ^{1}H NMR spectra

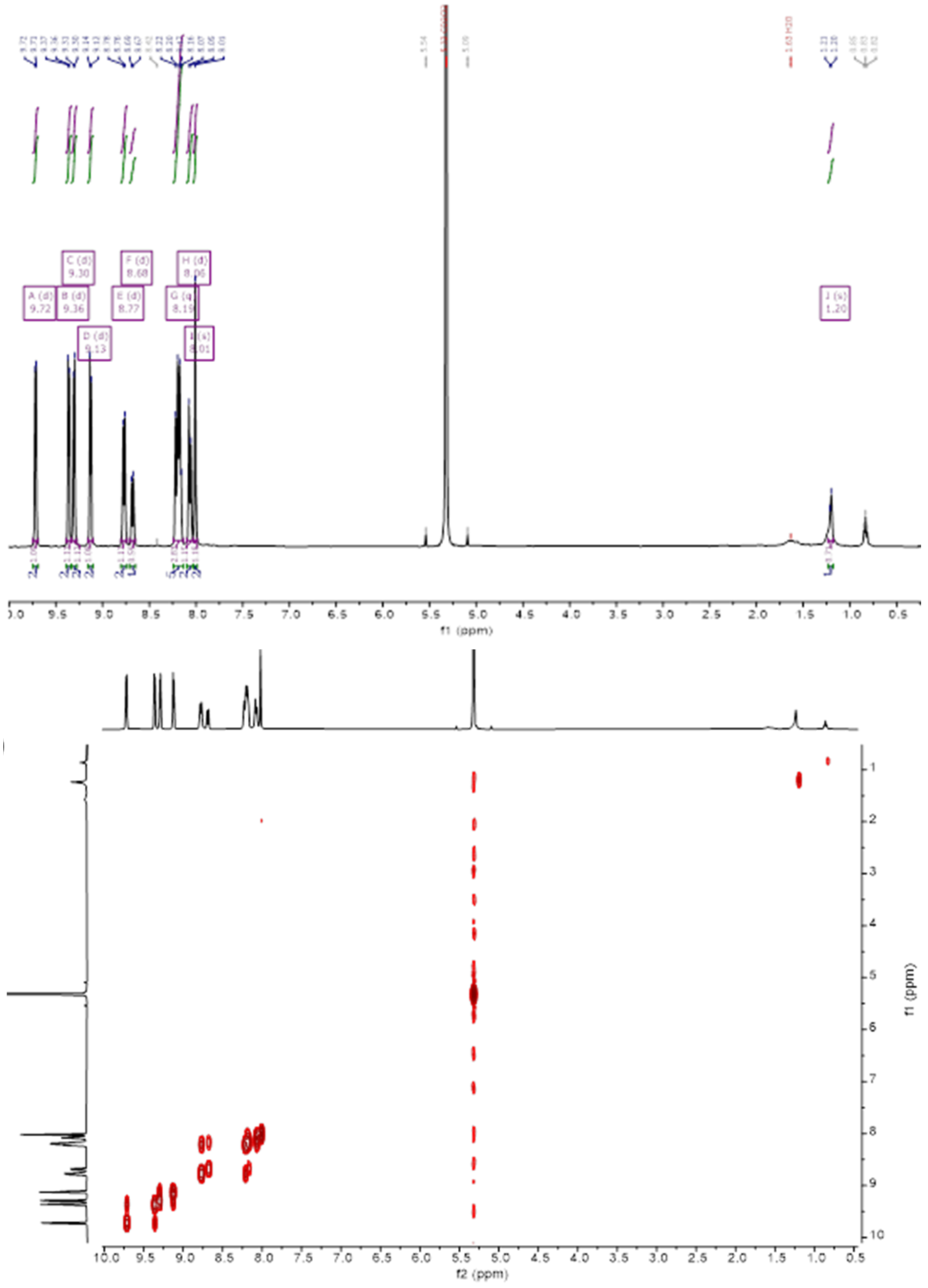

Figure S1. ^{1}H NMR spectra Re[T*p*CF_3PC](S) in $CDCl_3$ at 243 K: 1D spectrum (top) and ^{1}H-^{1}H COSY (bottom).

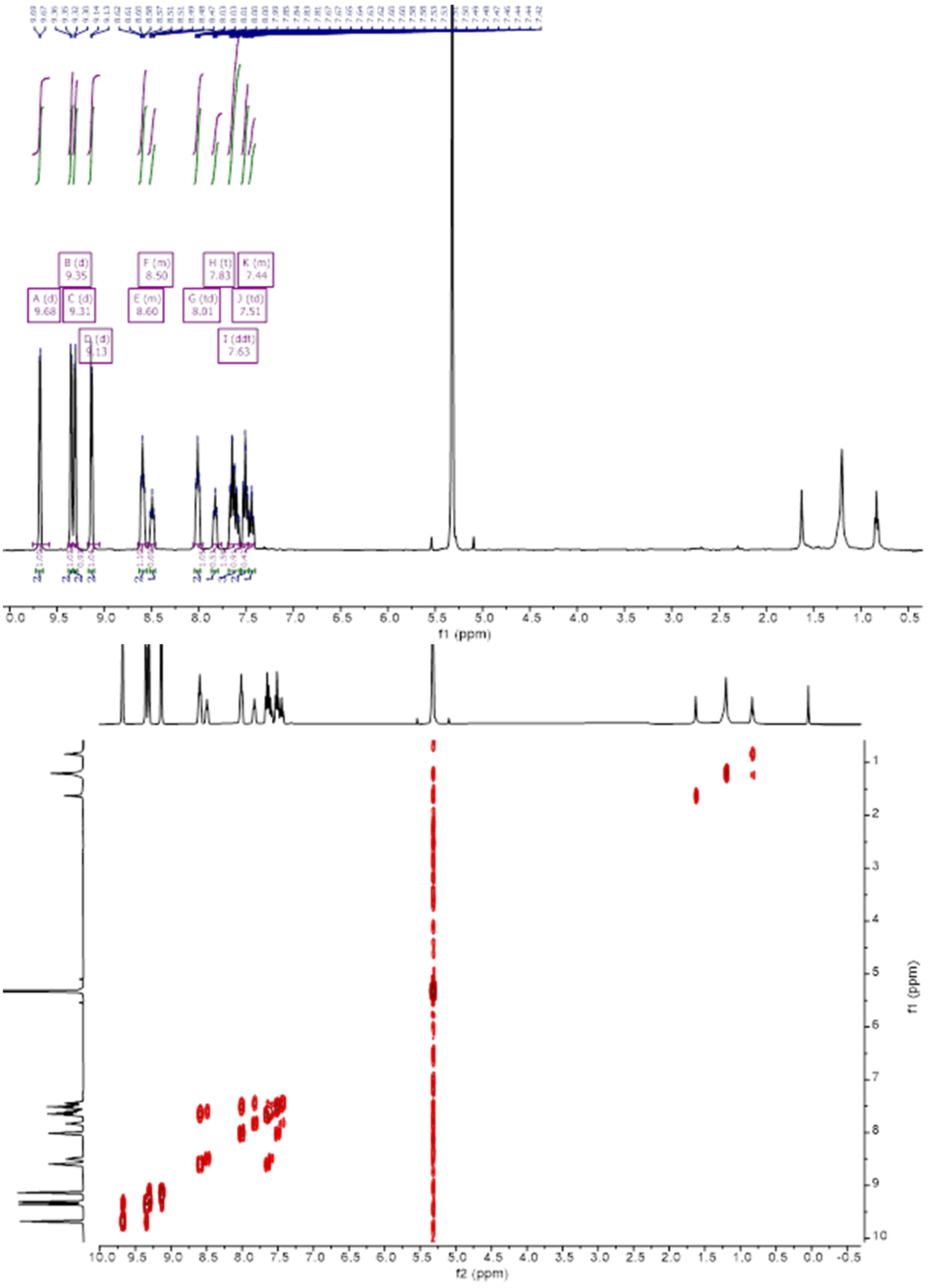

Figure S2. ${}^{1}H$ NMR spectra of Re[T*p*FPC](S) in CD_2Cl_2 at 243 K: 1D spectrum (top) and ${}^{1}H$-${}^{1}H$ COSY (bottom).

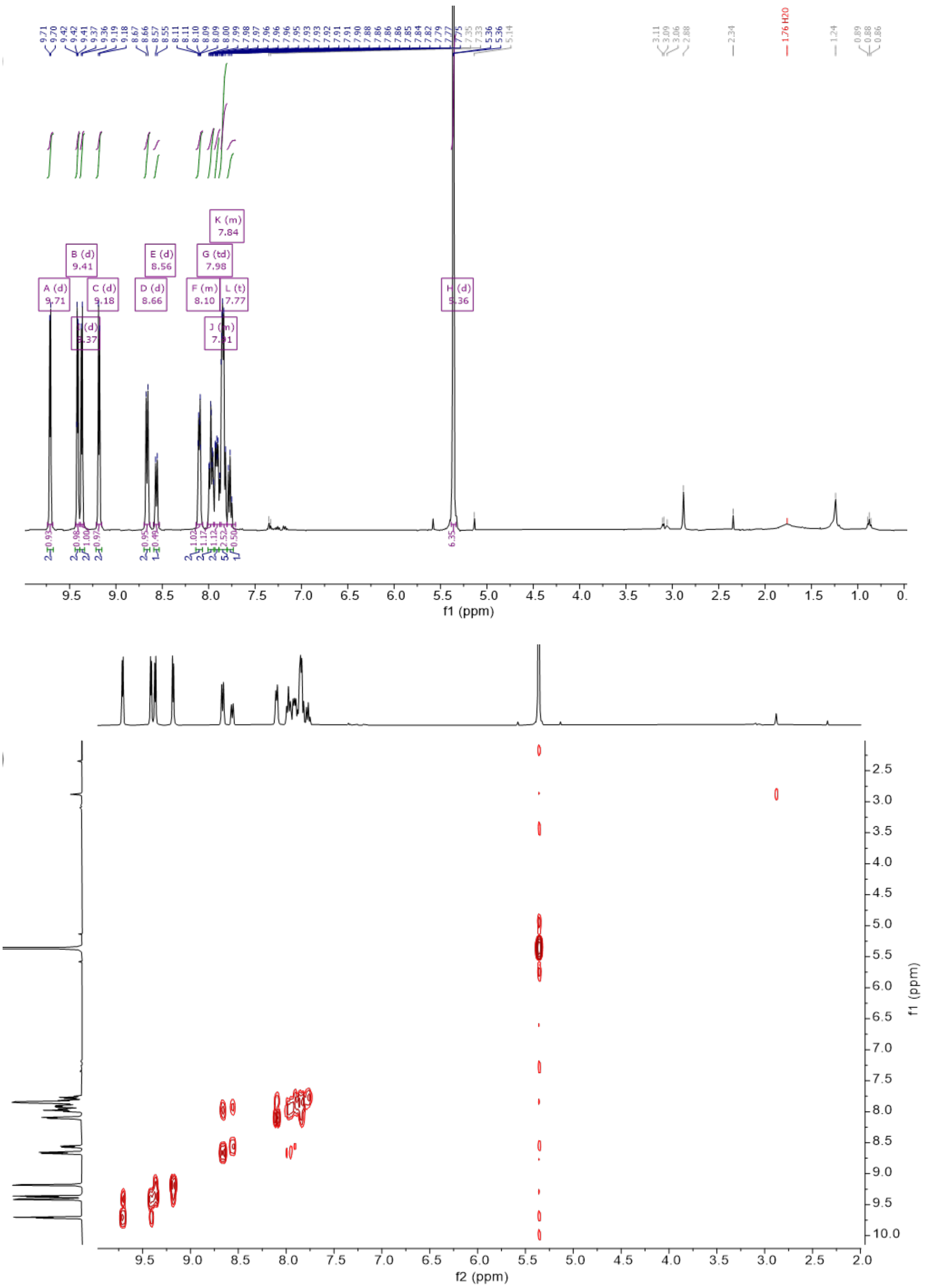

Figure S3. ^{1}H NMR spectra of Re[TPC](S) in CD_2Cl_2 at 243 K: 1D spectrum (top) and ^{1}H-^{1}H COSY (bottom).

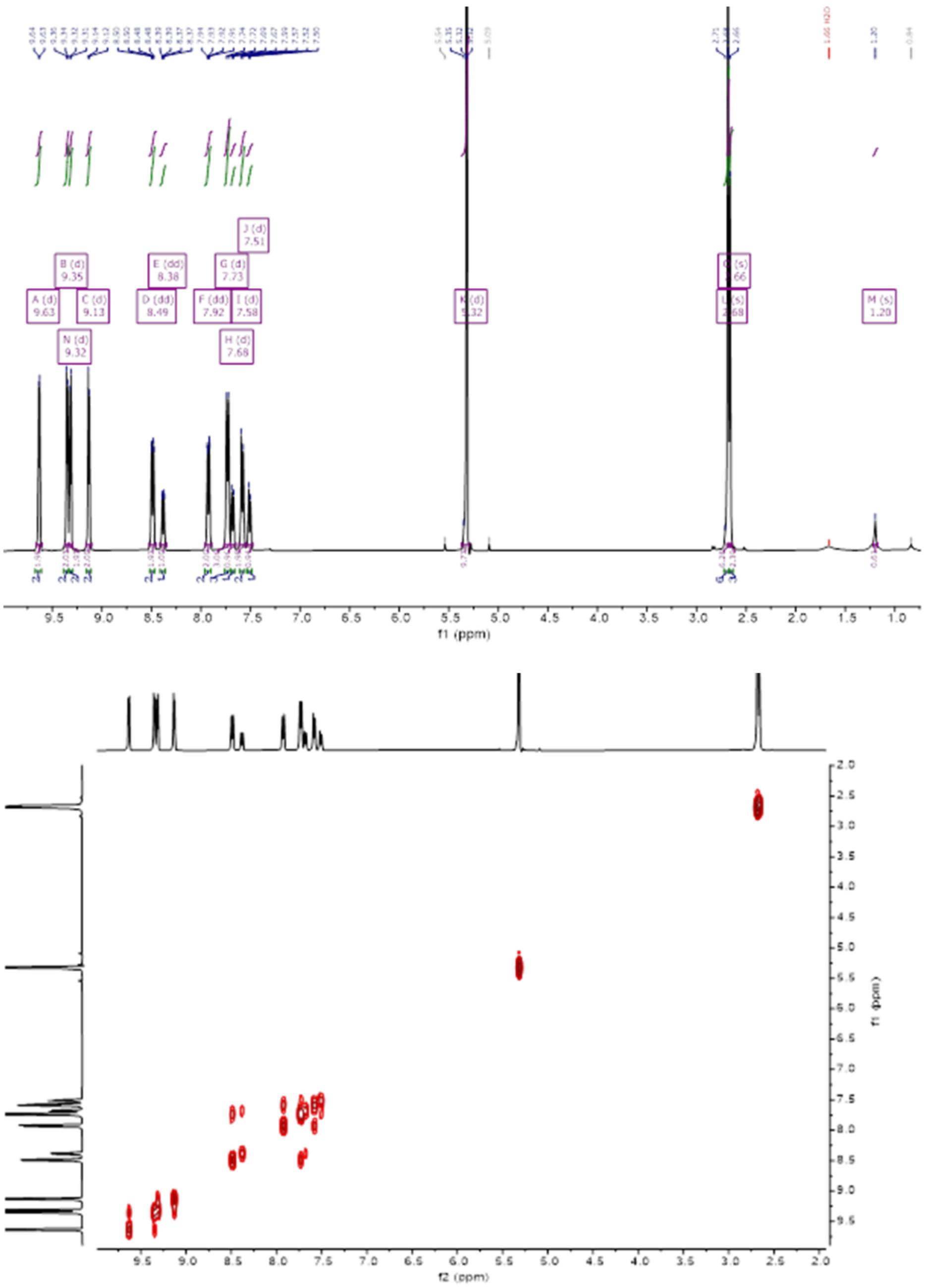

Figure S4. ^{1}H NMR spectra of Re[T*p*CH$_3$PC](S) in CD_2Cl_2 at 243 K: 1D spectrum (top) and ^{1}H-^{1}H COSY (bottom).

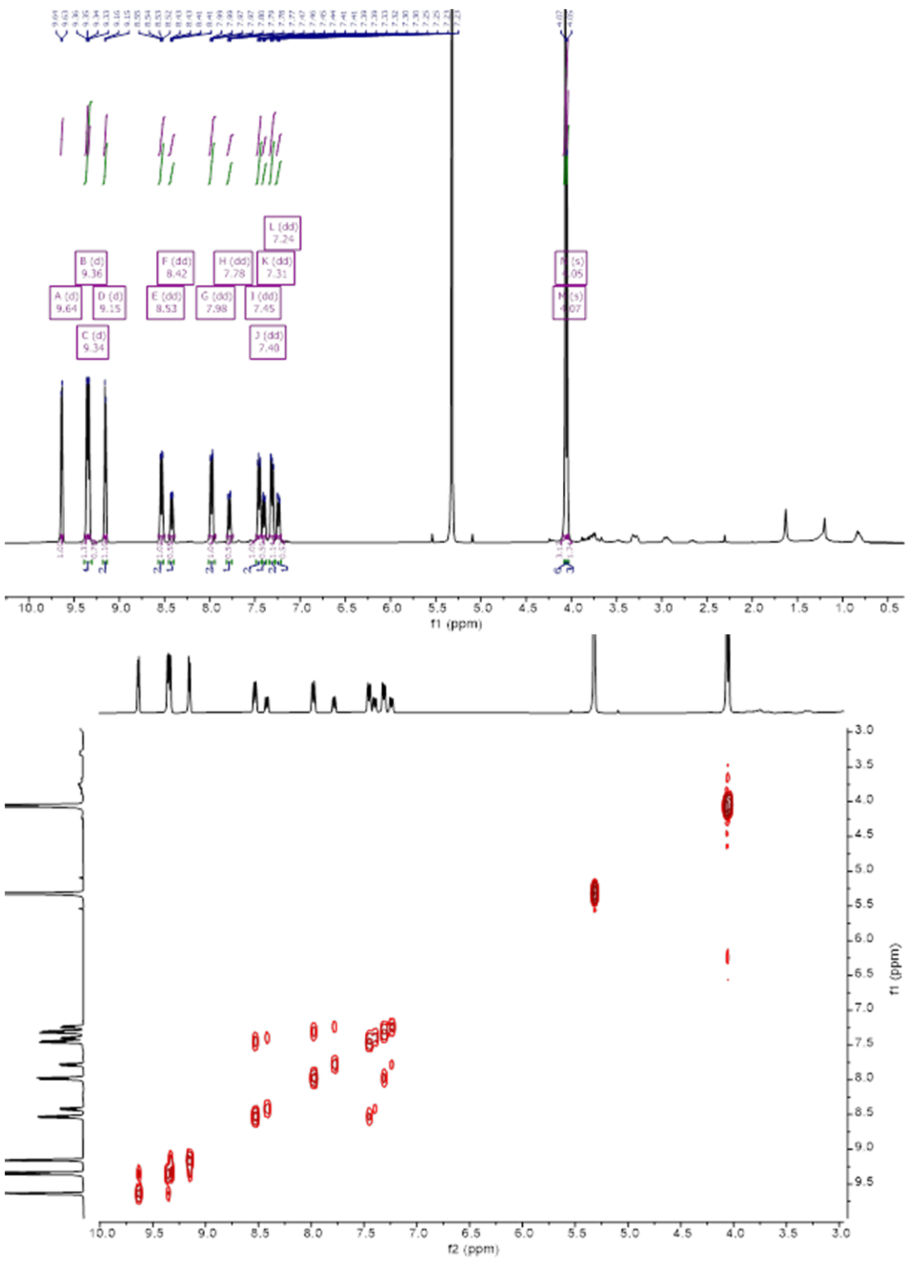

Figure S5. ^{1}H NMR spectra of Re[T*p*OCH$_3$PC](S) in CD_2Cl_2 at 243 K: 1D spectrum (top) and ^{1}H-^{1}H COSY (bottom).

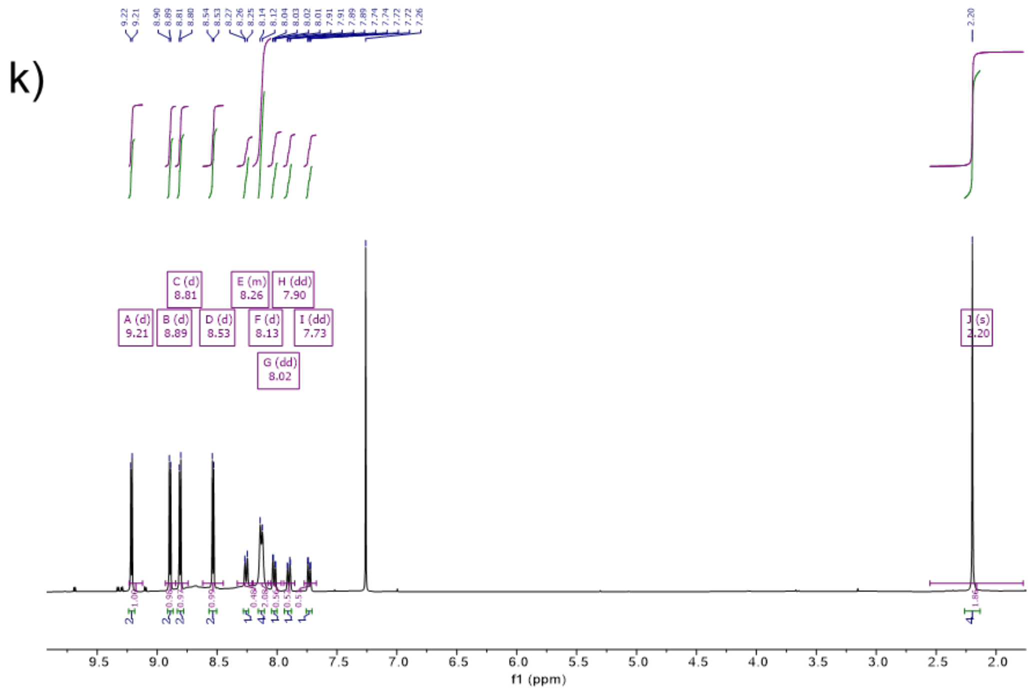

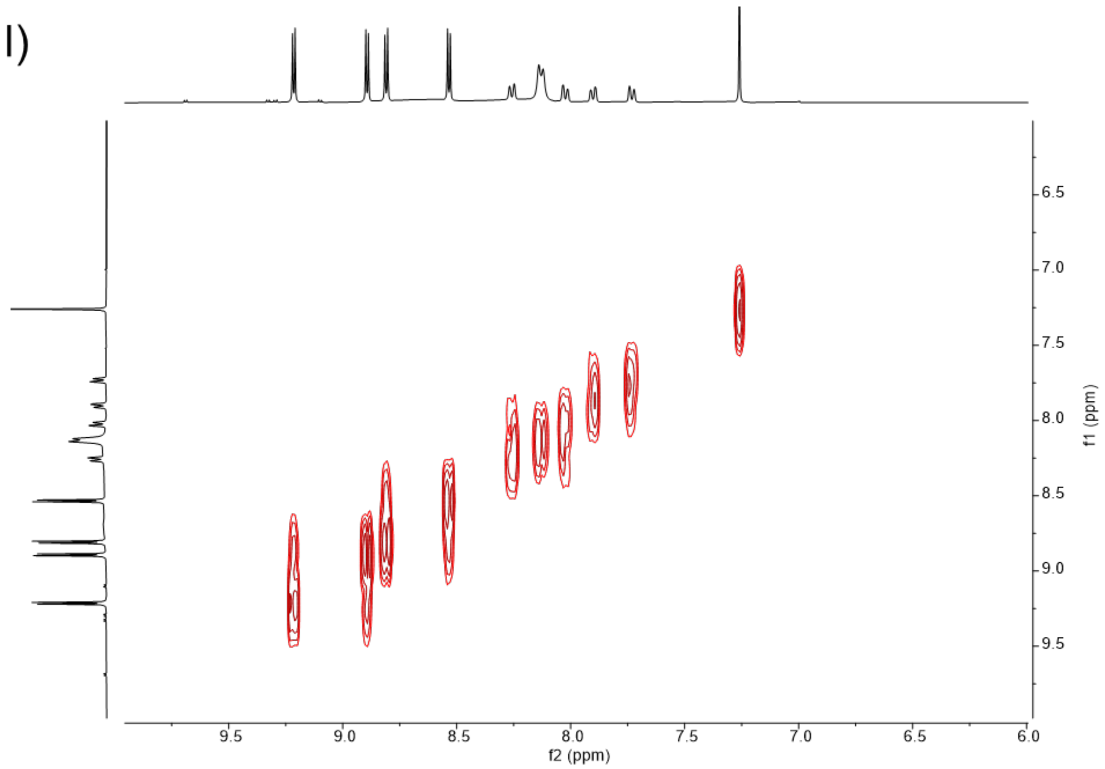

Figure S6. ^{1}H NMR spectra of Re[T*p*CF_3PC]($S_2C_2H_4$) in $CDCl_3$ at 298 K: 1D spectrum (top) and ^{1}H-^{1}H COSY (bottom).

m)

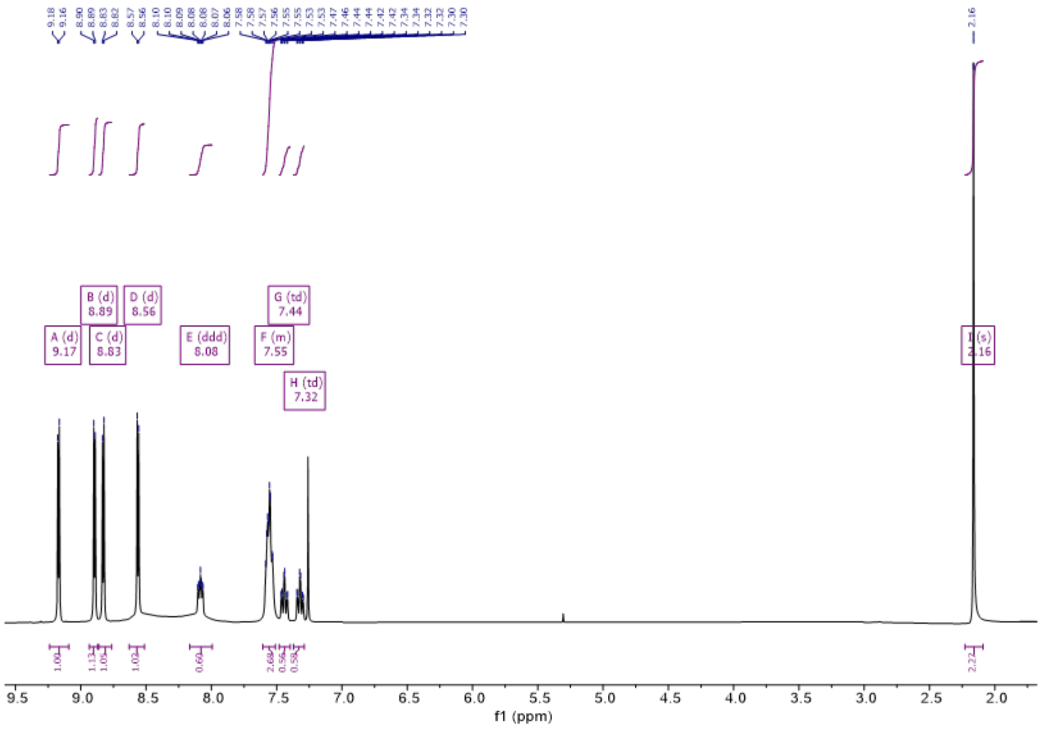

n)

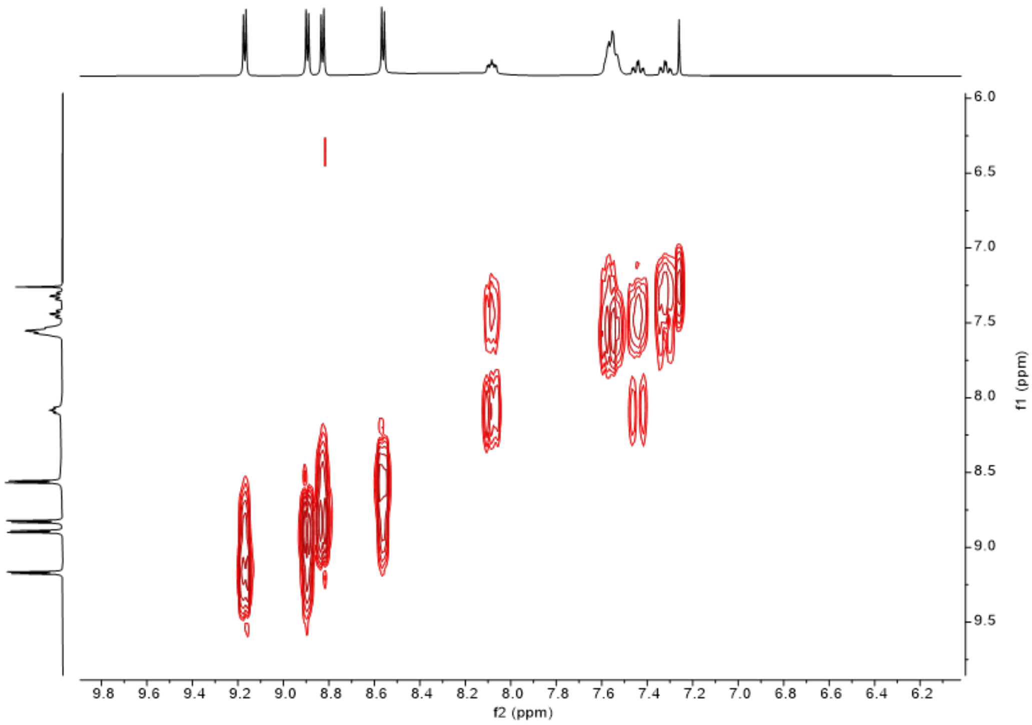

Figure S7. ^{1}H NMR spectra of Re[T*p*FPC]($S_2C_2H_4$) in $CDCl_3$ at 298 K: 1D spectrum (top) and ^{1}H-^{1}H COSY (bottom).

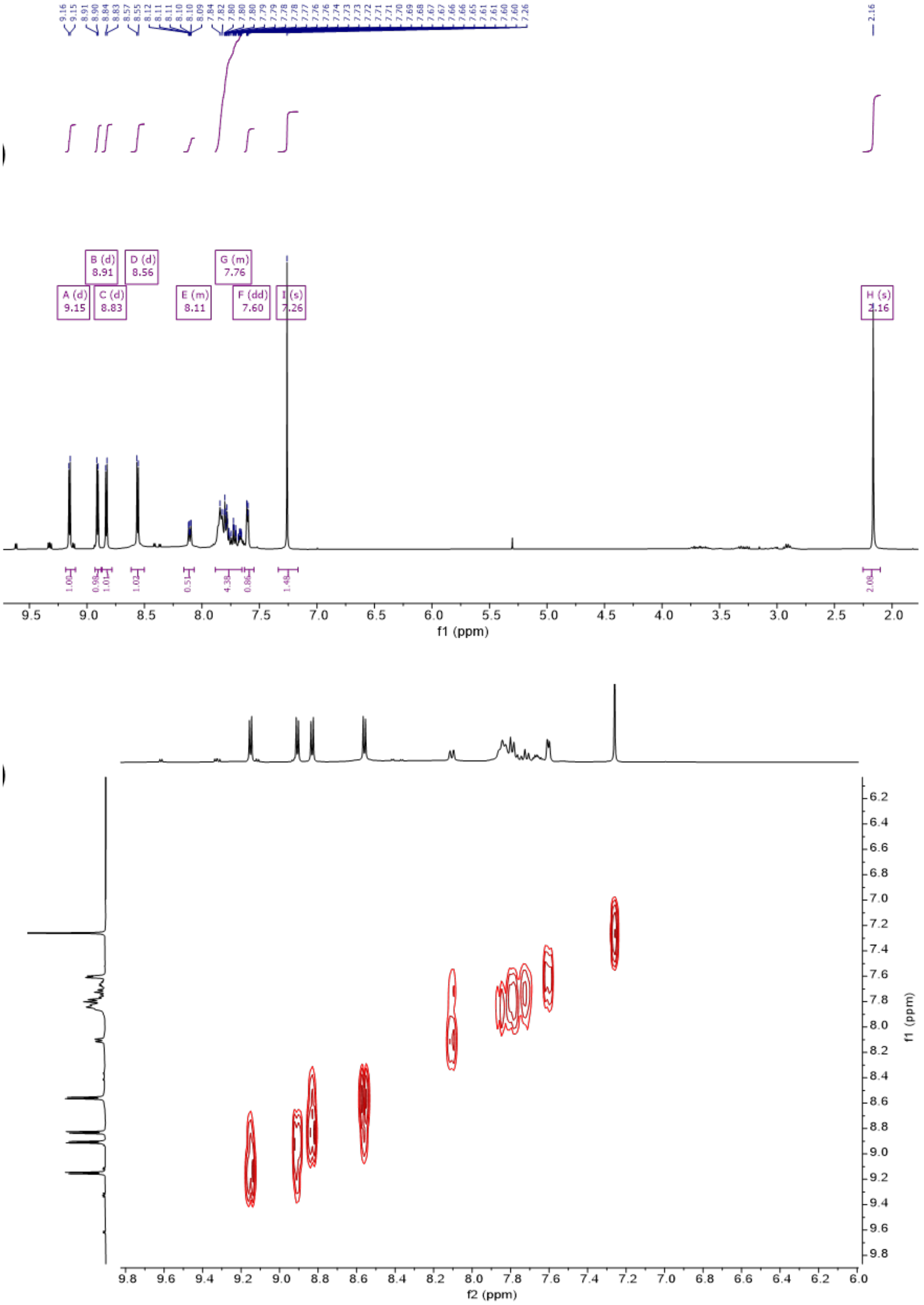

Figure S8. ^{1}H NMR spectrum (**o**) and ^{1}H-^{1}H COSY spectrum (**p**) of Re[TPC]($S_2C_2H_4$) in $CDCl_3$ at 298 K: 1D spectrum (top) and ^{1}H-^{1}H COSY (bottom).

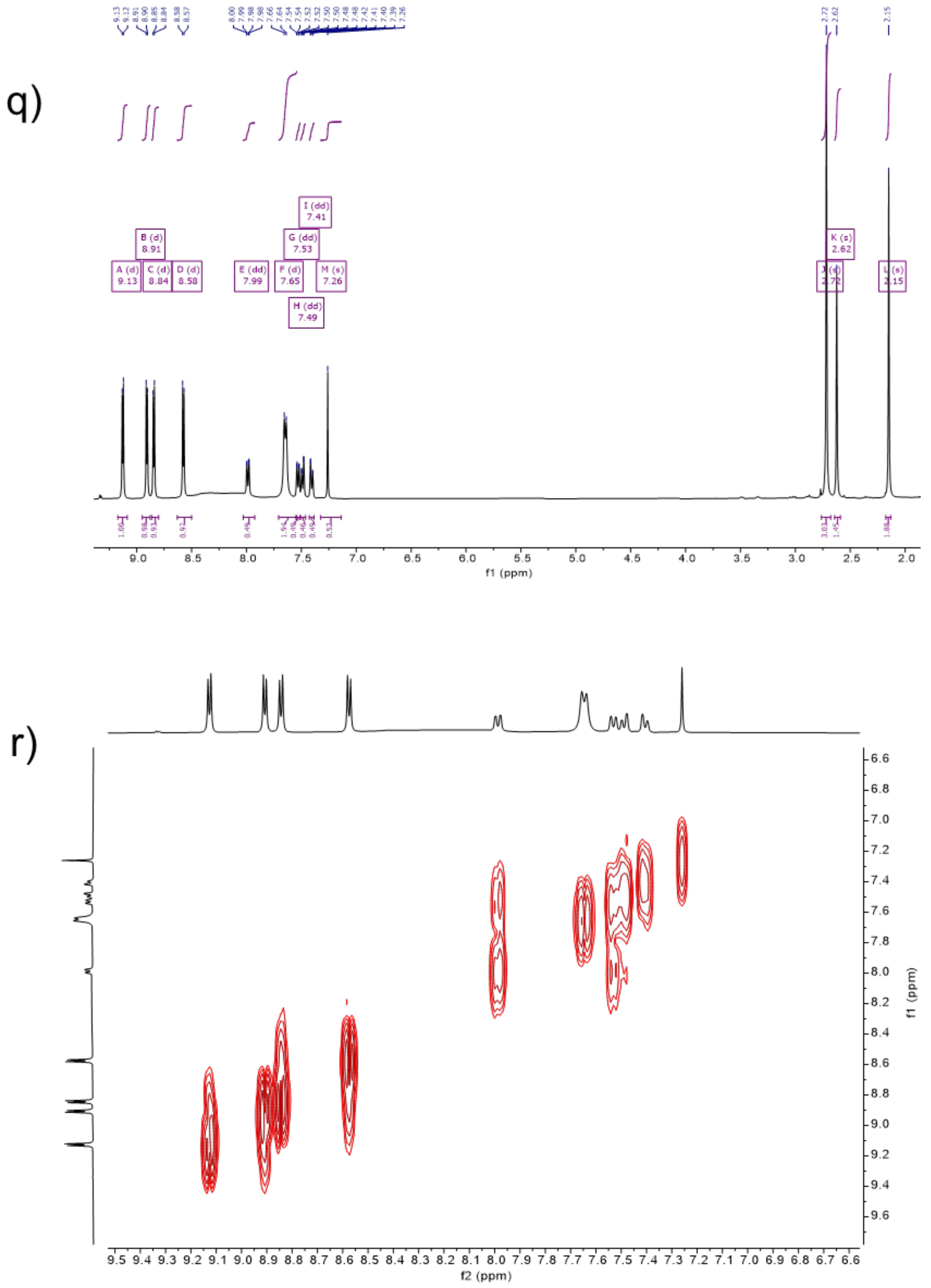

Figure S9. ^{1}H NMR spectra of Re[T*p*CH$_3$PC](S$_2$C$_2$H$_4$) in CDCl$_3$ at 298 K: 1D spectrum (top) and ^{1}H-^{1}H COSY (bottom).

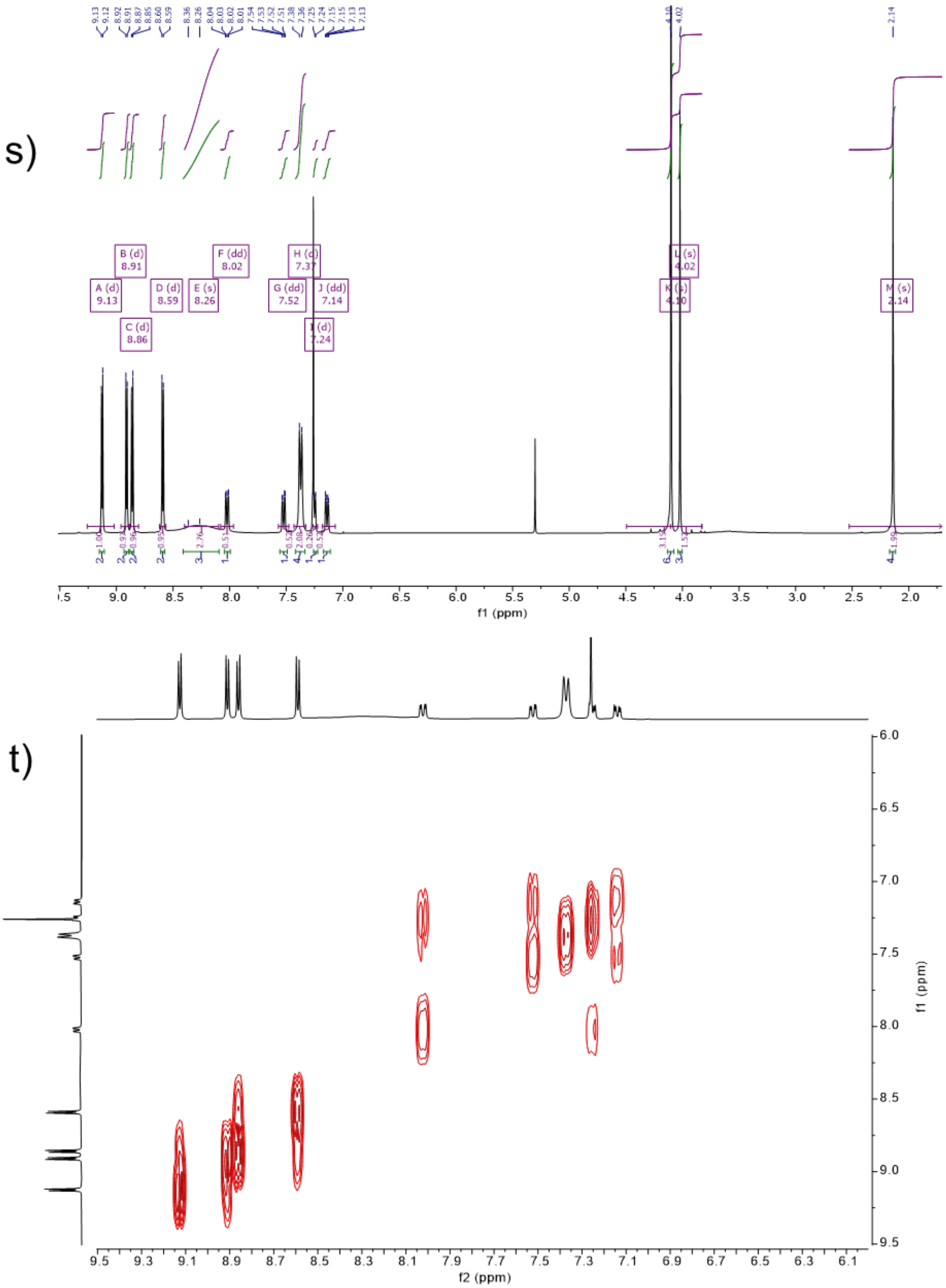

Figure S10. ^{1}H NMR spectra of Re[T*p*OCH$_3$PC]($S_2C_2H_4$) in $CDCl_3$ at 298 K: 1D spectrum (top) and ^{1}H-^{1}H COSY (bottom).

B. Electrospray ionization mass spectra

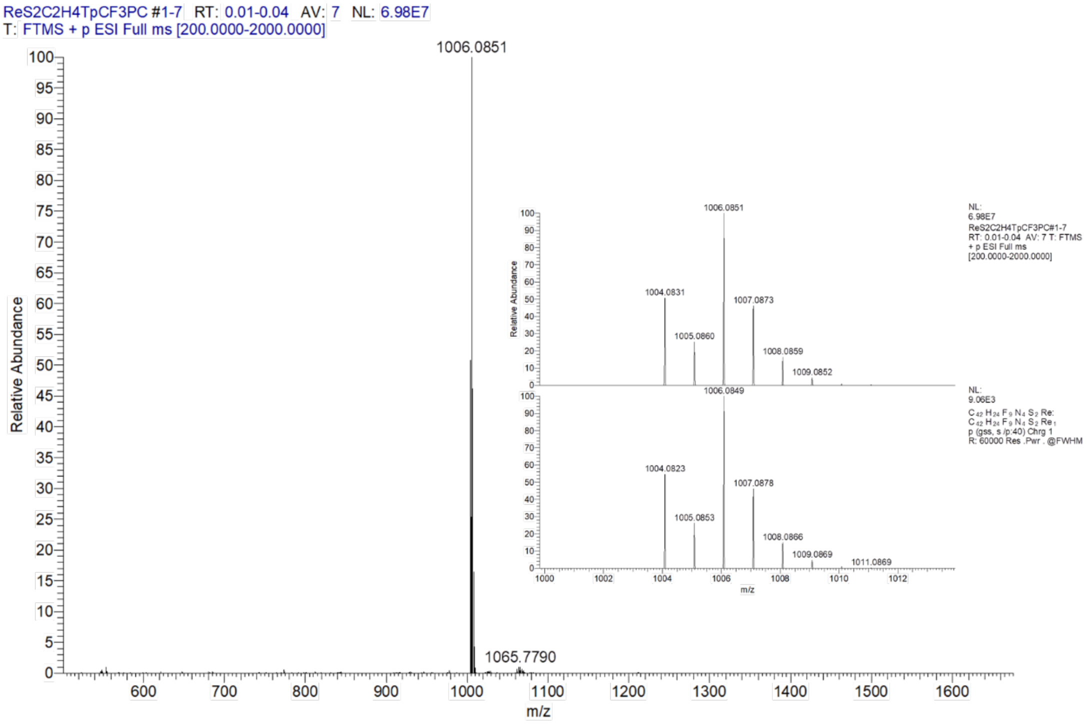

Figure S11. HR-ESI-MS of Re[T*p*CF_3PC]($S_2C_2H_4$). Inset: experimental molecular ion peak and simulation.

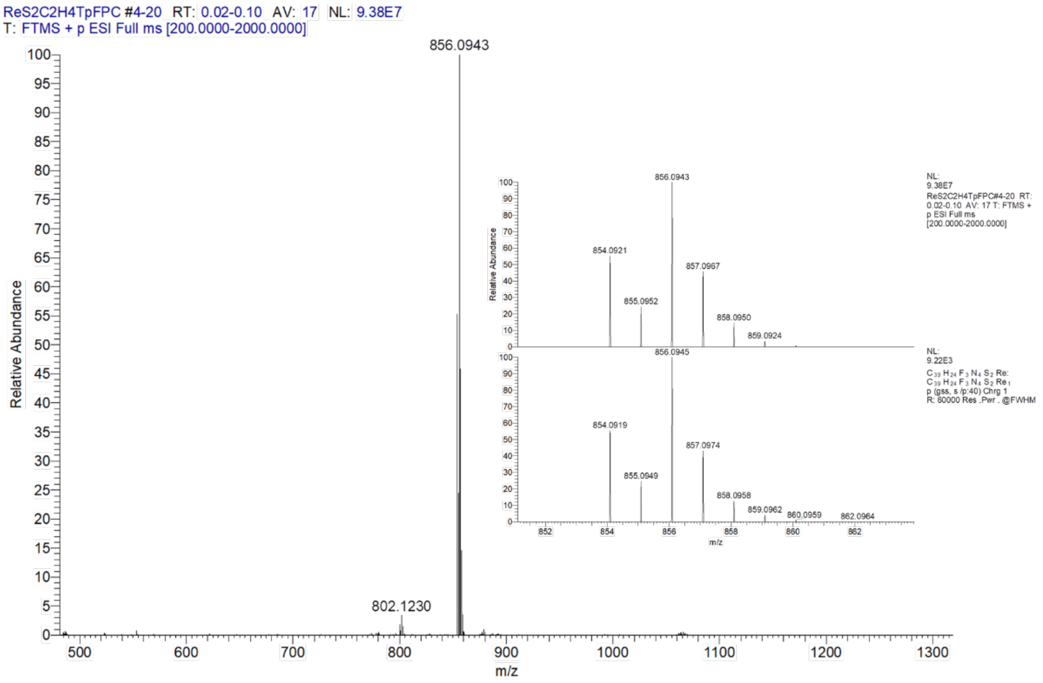

Figure S12. HRESI-MS of Re[T*p*FPC]($S_2C_2H_4$). Inset: experimental molecular ion peak and simulation.

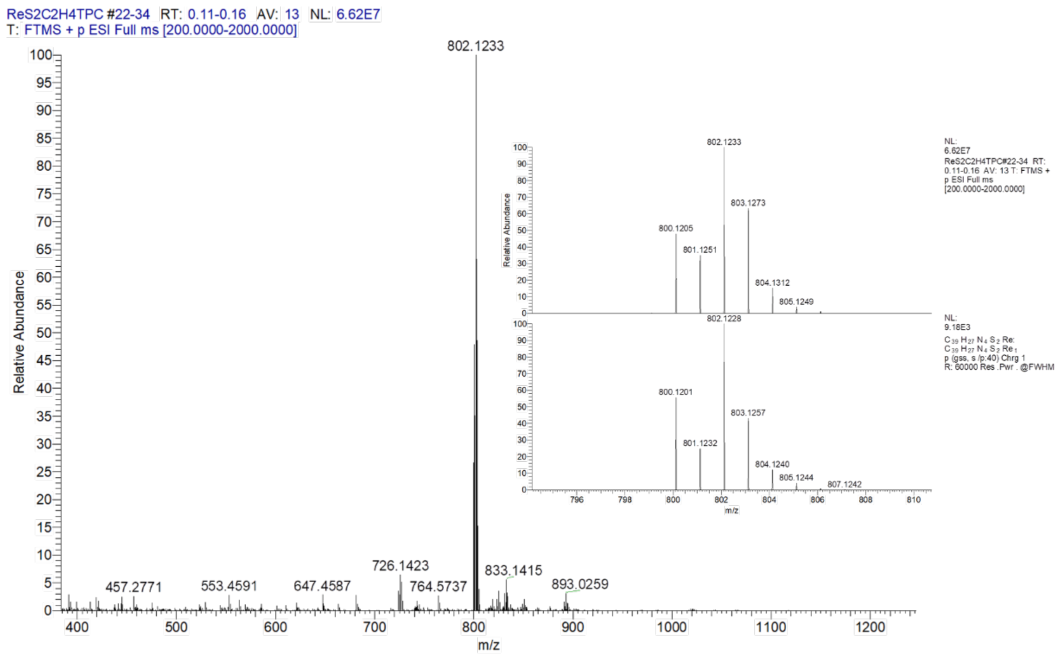

Figure S13. HRESI-MS of Re[TPC]($S_2C_2H_4$). Inset: experimental molecular ion peak and simulation.

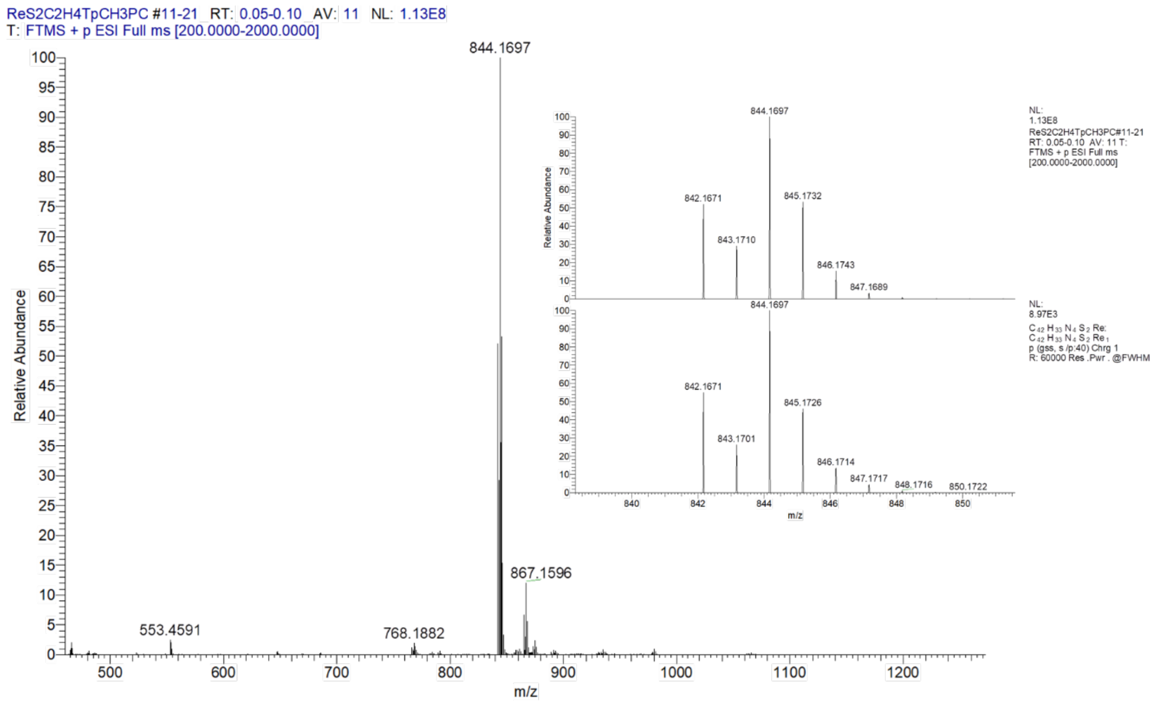

Figure S14. HRESI-MS of Re[T*p*CH_3PC]($S_2C_2H_4$). Inset: experimental molecular ion peak and simulation.

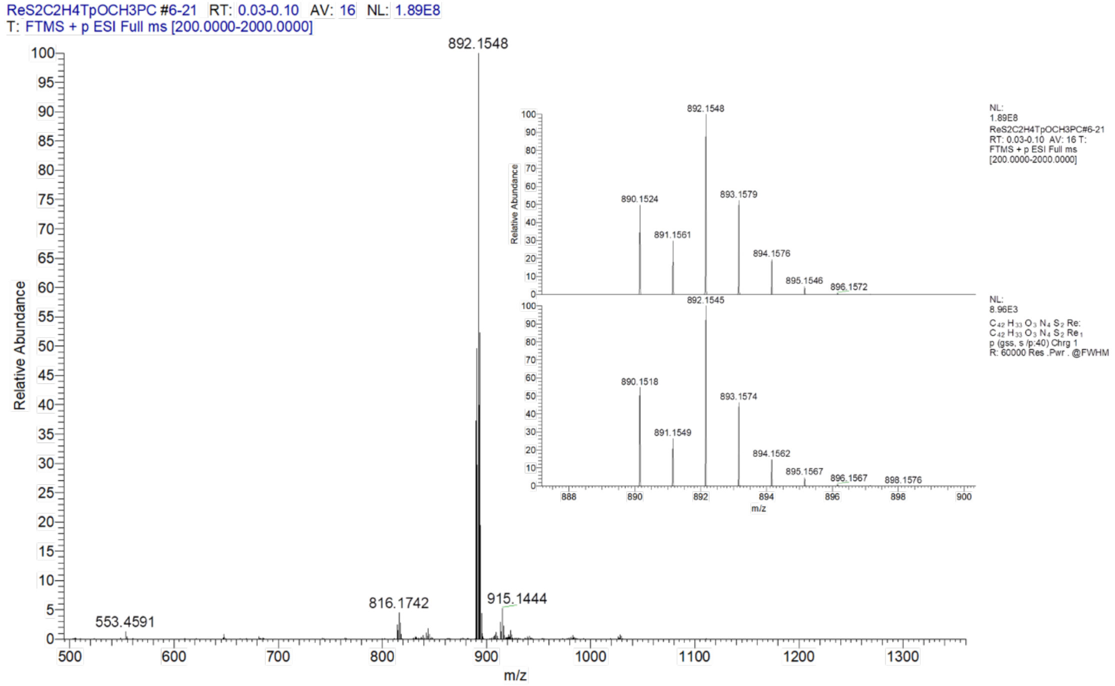

Figure S15. HRESI-MS of Re[T*p*OCH$_3$PC]($S_2C_2H_4$). Inset: experimental molecular ion peak and simulation.

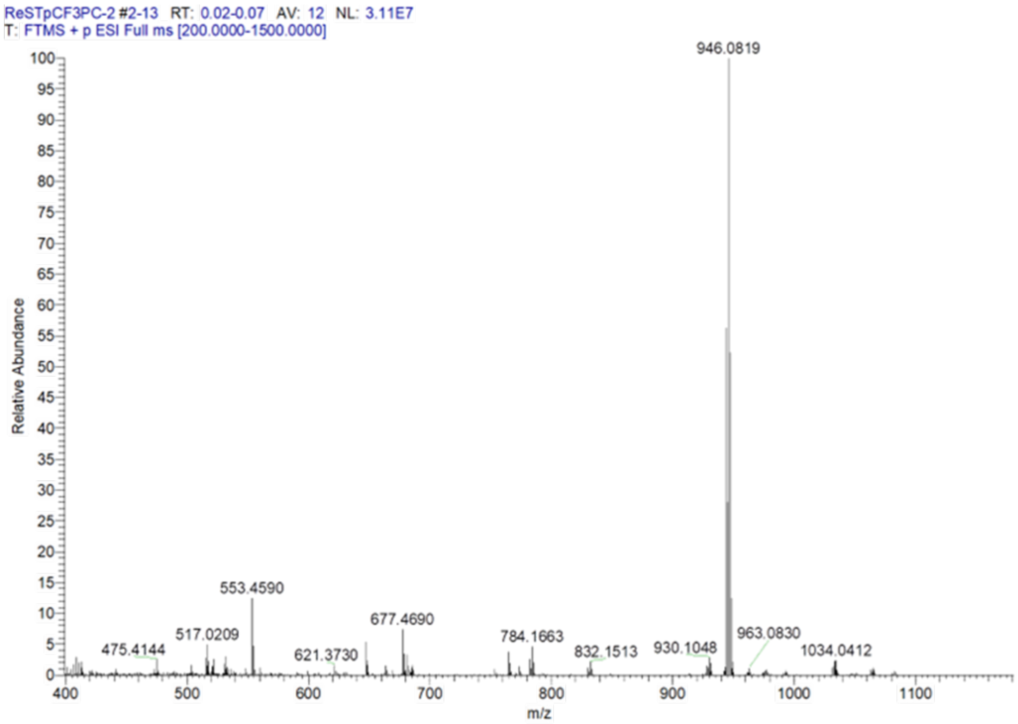

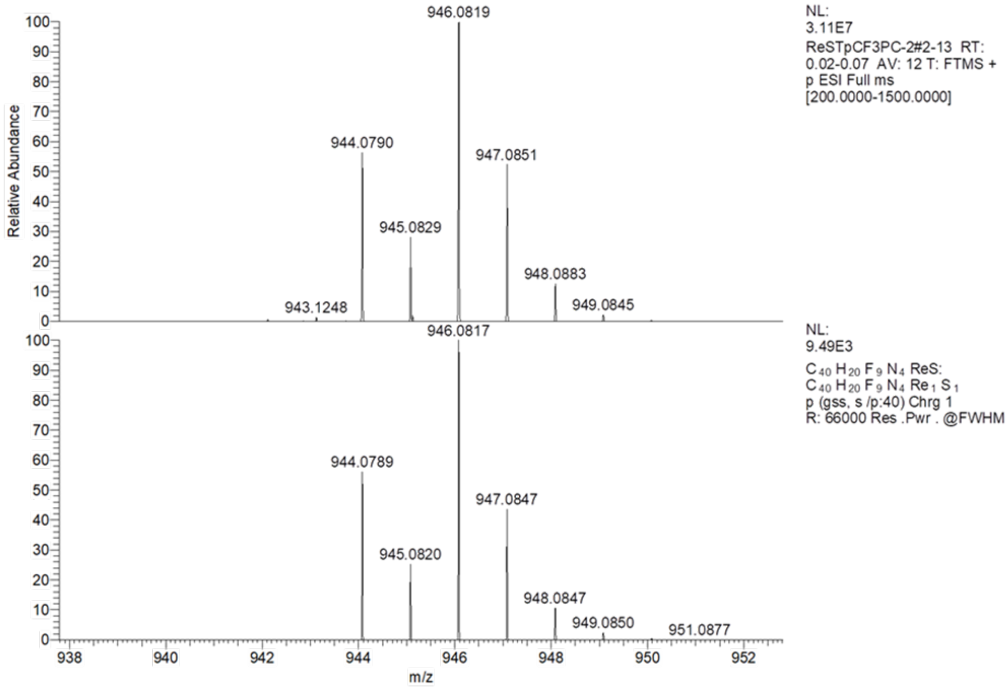

Figure S16. Above: HR-ESI-MS of Re[T*p*CF_3PC](S). Below: experimental molecular ion peak and simulation.

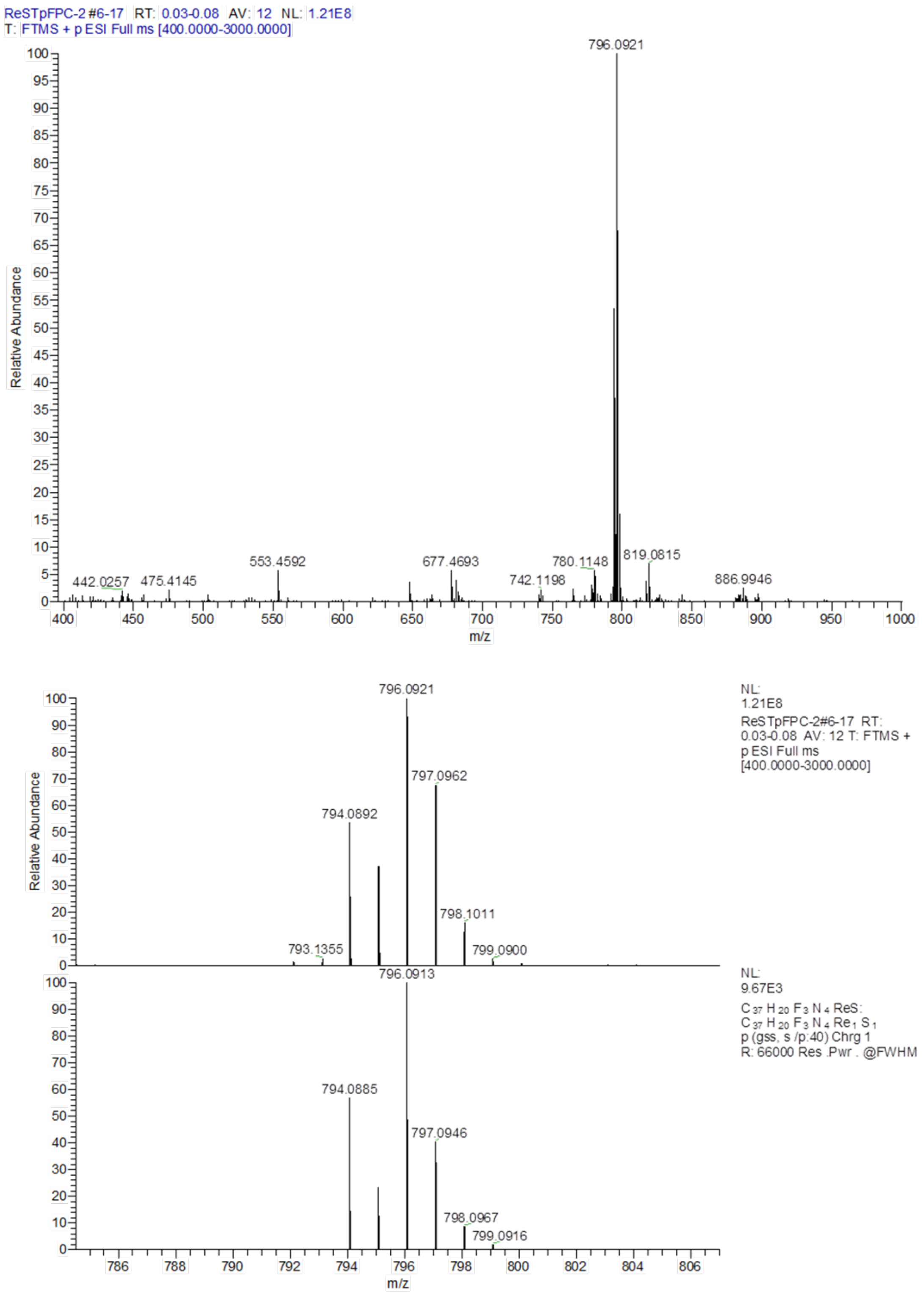

Figure S17. Above: HR-ESI-MS of Re[T*p*FPC](S). Below: experimental molecular ion peak and simulation.

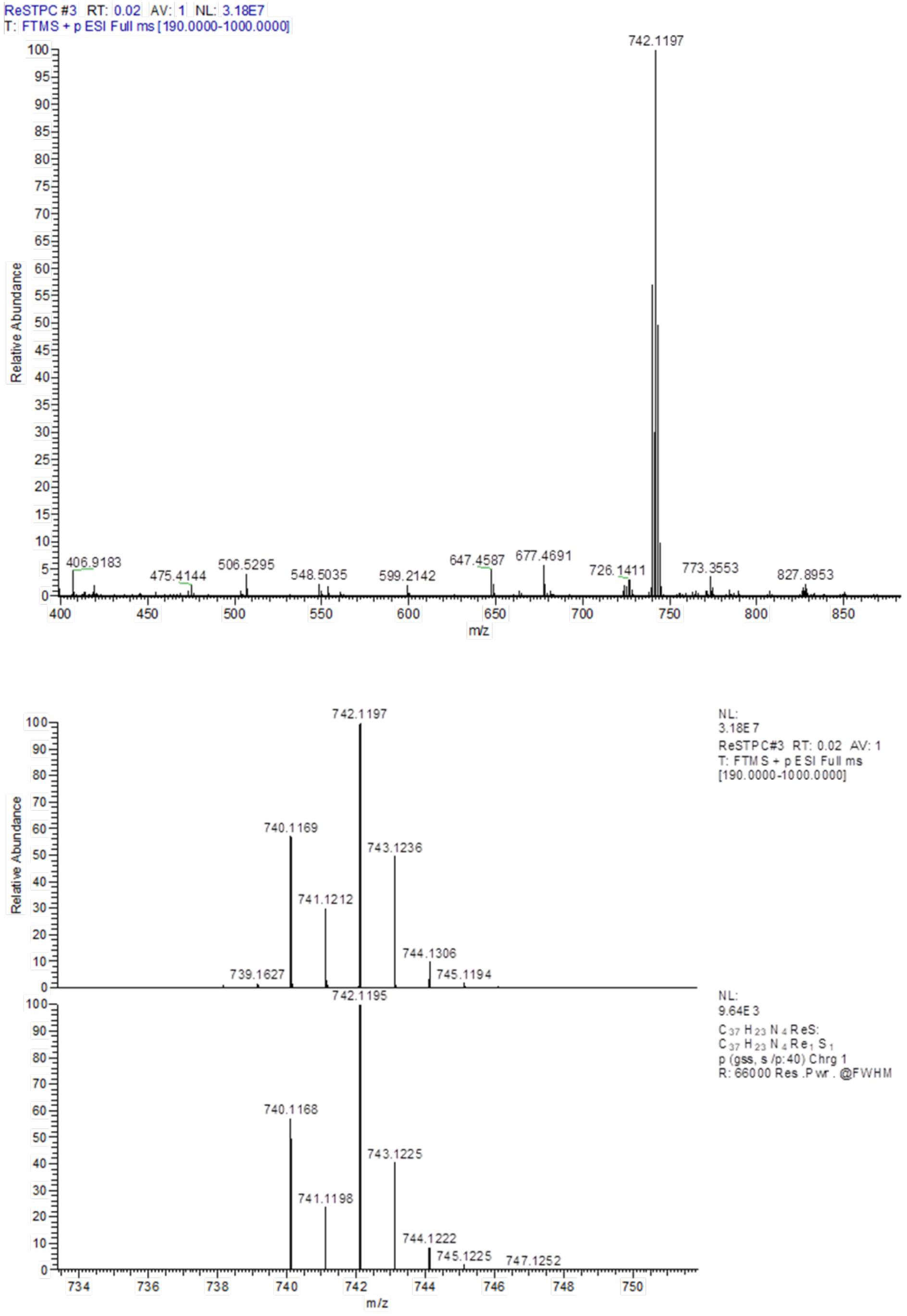

Figure S18. Above: HR-ESI-MS of Re[TPC](S). Below: experimental molecular ion peak and simulation.

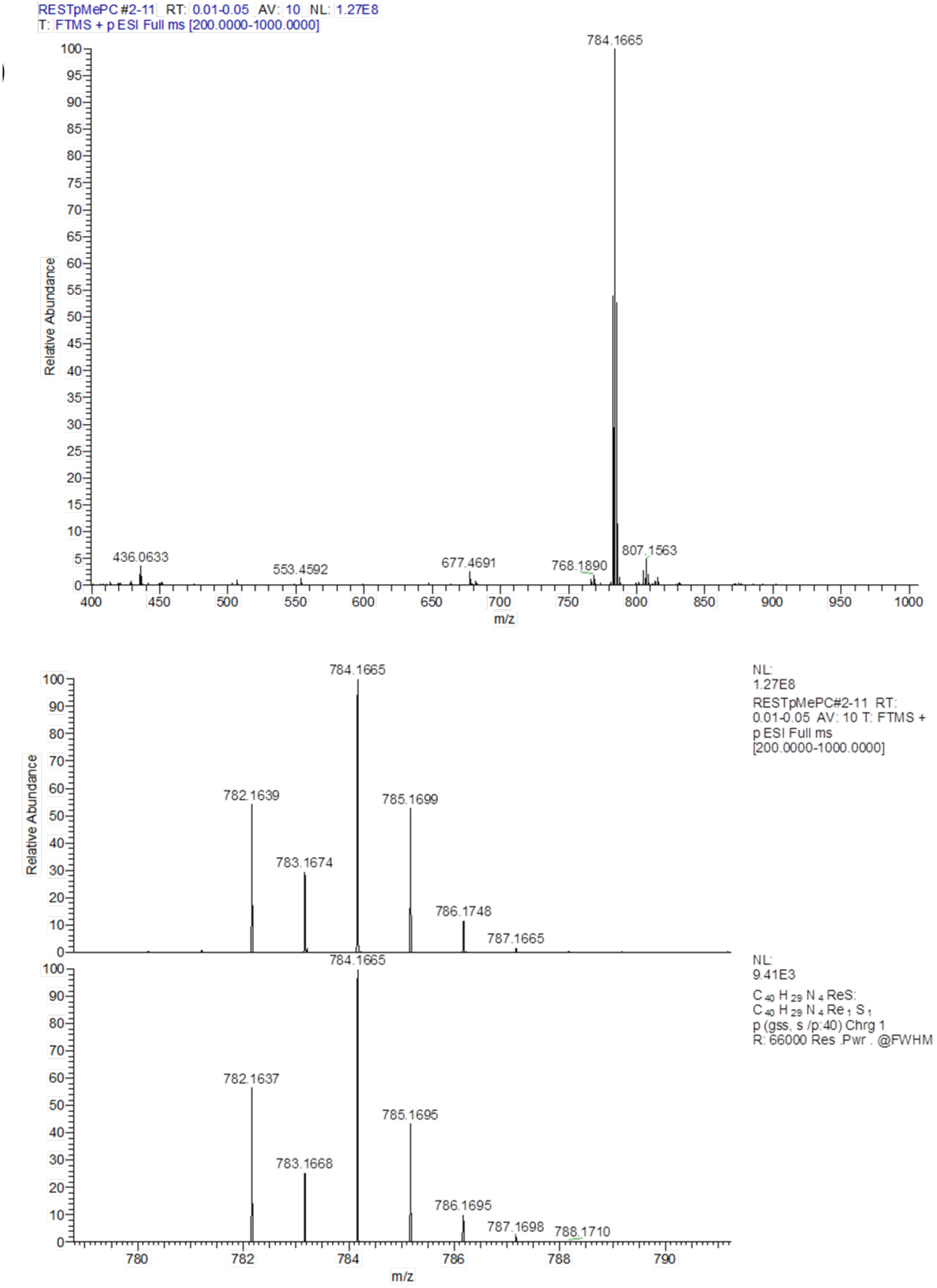

Figure S19. Above: HR-ESI-MS of Re[T*p*CH_3PC](S)]. Below: experimental molecular ion peak and simulation.

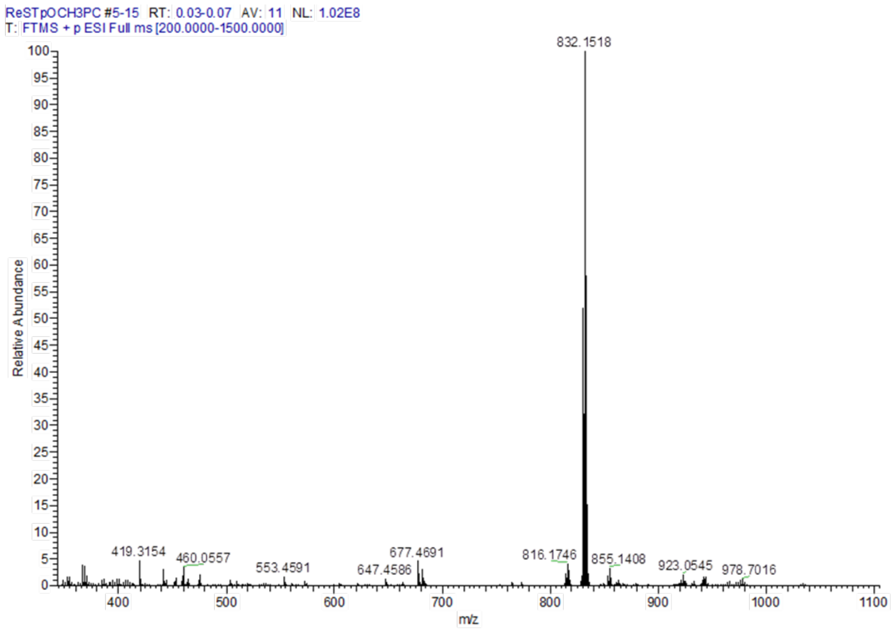

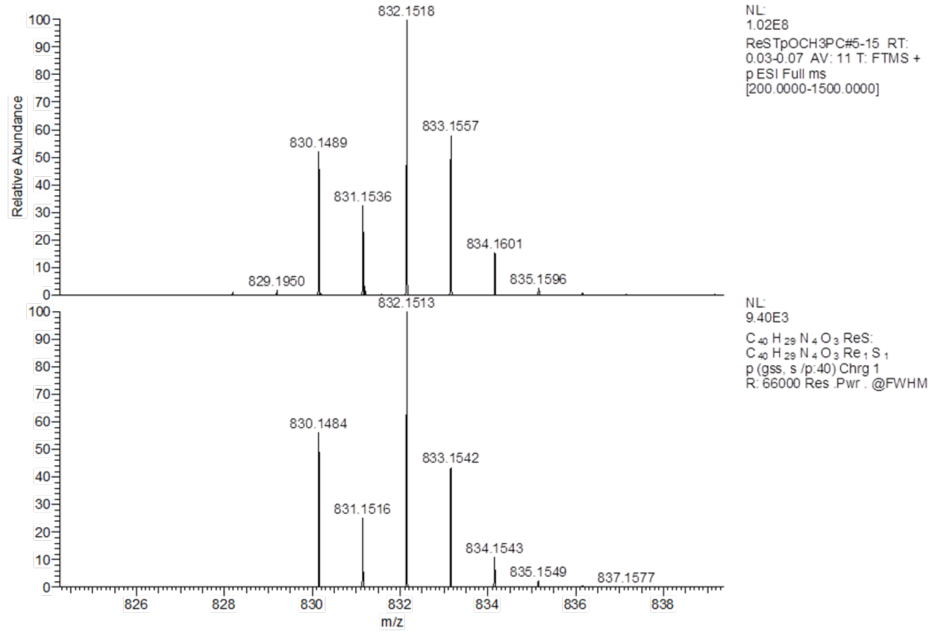

Figure S20. Above: HR-ESI-MS of Re[T*p*OCH$_3$PC](S)]. Below: experimental molecular ion peak and simulation.

C. OLYP-D3 optimized coordinates (Å)

Re[TPC](O)

Re	-0.292107000	-0.886267000	0.040193000
O	-0.813769000	-2.480327000	-0.038394000
N	1.277904000	-0.612831000	-1.195241000
N	-1.407518000	0.205257000	-1.231201000
N	-1.247855000	0.145529000	1.453764000
N	1.062319000	-0.658543000	1.484497000
C	2.607658000	-0.986395000	-0.913633000
C	3.284527000	-1.148824000	-2.158799000
C	2.409194000	-0.845495000	-3.172421000
C	1.160924000	-0.485272000	-2.584323000
C	-0.003824000	-0.081689000	-3.255181000
C	-1.191388000	0.294073000	-2.606650000
C	-2.348223000	0.873870000	-3.206111000
C	-3.250711000	1.146160000	-2.206740000
C	-2.678420000	0.743389000	-0.963181000
C	-3.237779000	0.945593000	0.311541000
C	-2.515061000	0.696863000	1.491039000
C	-2.813343000	0.968762000	2.858169000
C	-1.699615000	0.634456000	3.623643000
C	-0.719057000	0.131740000	2.743125000
C	0.615966000	-0.314339000	2.758127000
C	1.702653000	-0.434687000	3.649528000
C	2.811482000	-0.821565000	2.901197000
C	2.412855000	-0.934600000	1.537690000
C	3.163040000	-1.151143000	0.368386000
C	4.594349000	-1.509722000	0.515377000
C	4.961977000	-2.632282000	1.272811000
C	6.303182000	-2.975830000	1.432868000
C	7.302012000	-2.198921000	0.843053000
C	6.948446000	-1.073389000	0.096006000
C	5.607031000	-0.731187000	-0.064738000
C	0.014872000	-0.024431000	-4.741410000
C	0.904533000	0.812972000	-5.428159000
C	0.906929000	0.858083000	-6.821690000
C	0.016852000	0.067737000	-7.551424000
C	-0.875701000	-0.767782000	-6.877250000
C	-0.876106000	-0.812475000	-5.484086000
C	-4.613223000	1.492360000	0.409252000
C	-5.690536000	0.805785000	-0.169858000
C	-6.988563000	1.302799000	-0.068760000
C	-7.231539000	2.497847000	0.611057000
C	-6.165482000	3.193451000	1.184706000
C	-4.867407000	2.695082000	1.084306000
H	4.182496000	-3.237236000	1.726843000
H	6.568541000	-3.855655000	2.014757000
H	8.348458000	-2.467587000	0.966356000
H	7.720197000	-0.455604000	-0.357728000
H	5.331730000	0.151090000	-0.634516000
H	-1.568350000	-1.461810000	-4.956038000
H	1.590785000	1.432737000	-4.858536000
H	1.600088000	1.517928000	-7.338721000
H	0.017969000	0.103633000	-8.638258000

H	-1.570698000	-1.389544000	-7.437191000
H	-4.036310000	3.239463000	1.522508000
H	-6.344202000	4.130484000	1.707370000
H	-8.244333000	2.885499000	0.691533000
H	-7.812828000	0.752282000	-0.516454000
H	-5.498626000	-0.126538000	-0.692828000
H	-4.216113000	1.619767000	-2.306193000
H	-2.454679000	1.075173000	-4.261628000
H	2.599876000	-0.876489000	-4.234423000
H	4.312720000	-1.462657000	-2.257210000
H	3.816987000	-0.981116000	3.262741000
H	1.677736000	-0.228133000	4.711660000
H	-1.591976000	0.748419000	4.694584000
H	-3.744791000	1.382551000	3.215271000

Re[TPC](S)

Re	-0.308871000	-0.930746000	0.024980000
S	-0.949055000	-2.910751000	-0.119863000
N	1.263846000	-0.653257000	-1.194971000
N	-1.419467000	0.172937000	-1.232215000
N	-1.279279000	0.059586000	1.451978000
N	1.030871000	-0.734168000	1.482914000
C	2.595618000	-1.012170000	-0.912459000
C	3.276868000	-1.169953000	-2.156023000
C	2.401190000	-0.872258000	-3.171451000
C	1.151624000	-0.515832000	-2.583765000
C	-0.008692000	-0.100303000	-3.252710000
C	-1.194938000	0.279134000	-2.606015000
C	-2.342172000	0.877979000	-3.205545000
C	-3.244202000	1.154030000	-2.206591000
C	-2.681682000	0.730261000	-0.965294000
C	-3.241897000	0.926704000	0.309587000
C	-2.535393000	0.638686000	1.487579000
C	-2.838891000	0.901816000	2.856164000
C	-1.734754000	0.545037000	3.623587000
C	-0.755655000	0.037141000	2.742657000
C	0.579205000	-0.403284000	2.758255000
C	1.666089000	-0.511827000	3.652392000
C	2.782660000	-0.872465000	2.904372000
C	2.389078000	-0.980782000	1.537857000
C	3.148810000	-1.168671000	0.371389000
C	4.592295000	-1.475888000	0.517183000
C	5.005943000	-2.581734000	1.275220000
C	6.360478000	-2.869342000	1.434175000
C	7.325964000	-2.053616000	0.840929000
C	6.925932000	-0.946564000	0.089644000
C	5.571529000	-0.659685000	-0.068898000
C	0.013850000	-0.040770000	-4.738545000
C	0.907034000	0.792886000	-5.424561000
C	0.916292000	0.830066000	-6.818460000
C	0.030672000	0.034614000	-7.548049000
C	-0.865357000	-0.797325000	-6.873906000
C	-0.873516000	-0.833322000	-5.480647000
C	-4.598045000	1.521361000	0.410841000
C	-5.701345000	0.874633000	-0.164498000

C	-6.979773000	1.420460000	-0.059765000
C	-7.175499000	2.625499000	0.617963000
C	-6.082538000	3.281100000	1.187827000
C	-4.804788000	2.733338000	1.085418000
H	4.252546000	-3.217305000	1.731170000
H	6.662616000	-3.735414000	2.018469000
H	8.383002000	-2.276966000	0.965749000
H	7.671159000	-0.298990000	-0.366857000
H	5.259137000	0.208655000	-0.641006000
H	-1.567907000	-1.479779000	-4.951633000
H	1.591551000	1.414379000	-4.854696000
H	1.611773000	1.486789000	-7.336219000
H	0.037366000	0.064296000	-8.635145000
H	-1.556831000	-1.422862000	-7.433926000
H	-3.952713000	3.246010000	1.521492000
H	-6.224293000	4.225209000	1.709215000
H	-8.172594000	3.051995000	0.699676000
H	-7.825984000	0.900518000	-0.503179000
H	-5.545853000	-0.064861000	-0.686690000
H	-4.202939000	1.641197000	-2.304604000
H	-2.442578000	1.087799000	-4.259981000
H	2.592865000	-0.902150000	-4.233274000
H	4.307607000	-1.476389000	-2.251754000
H	3.789970000	-1.016771000	3.267099000
H	1.635170000	-0.312050000	4.715523000
H	-1.630209000	0.649085000	4.695791000
H	-3.766747000	1.325519000	3.211083000
